# Increased intestinal permeability and downregulation of absorptive ion transporters *Nhe3*, *Dra*, and *Sglt1* contribute to diarrhea during *Clostridioides difficile* infection

**DOI:** 10.1080/19490976.2023.2225841

**Published:** 2023-06-23

**Authors:** F. Christopher Peritore-Galve, Izumi Kaji, Anna Smith, Lauren M. Walker, John A. Shupe, M. Kay Washington, Holly M. Scott Algood, Pradeep K. Dudeja, James R. Goldenring, D. Borden Lacy

**Affiliations:** a Department of Pathology, Microbiology, and Immunology, Vanderbilt University Medical Center, Nashville, TN, USA; b Vanderbilt Institute for Infection, Immunology, and Inflammation, Vanderbilt University Medical Center, Nashville, TN, USA; c Section of Surgical Sciences, Vanderbilt University Medical Center, Nashville, TN, USA; d Epithelial Biology Center, Vanderbilt University School of Medicine, Nashville, TN, USA; e Vanderbilt Vaccine Center, Vanderbilt University Medical Center, Nashville, TN, USA; f Department of Veterans Affairs, Tennessee Valley Healthcare System, Nashville, TN, USA; g Division of Gastroenterology and Hepatology, Department of Medicine, University of Illinois at Chicago, Chicago, IL, USA; h Department of Veterans Affairs, Jesse Brown Veterans Affairs Medical Center, Chicago, IL, USA; i Cell and Developmental Biology, Vanderbilt University School of Medicine, Nashville, TN, USA

**Keywords:** *Clostridioides difficile infection*, paracellular permeability, ion transport, toxins

## Abstract

**Background & Aim:**

*Clostridioides difficile* infection (CDI) is the leading cause of hospital-acquired diarrhea and pseudomembranous colitis. Two protein toxins, TcdA and TcdB, produced by *C. difficile* are the major determinants of disease. However, the pathophysiological causes of diarrhea during CDI are not well understood. Here, we investigated the effects of *C. difficile* toxins on paracellular permeability and apical ion transporters in the context of an acute physiological infection.

**Methods:**

We studied intestinal permeability and apical membrane transporters in female C57BL/6J mice. Üssing chambers were used to measure paracellular permeability and ion transporter function across the intestinal tract. Infected intestinal tissues were analyzed by immunofluorescence microscopy and RNA-sequencing to uncover mechanisms of transporter dysregulation.

**Results:**

Intestinal permeability was increased through the size-selective leak pathway *in vivo* during acute CDI in a 2-day-post infection model. Chloride secretory activity was reduced in the cecum and distal colon during infection by decreased CaCC and CFTR function, respectively. SGLT1 activity was significantly reduced in the cecum and colon, accompanied by ablated SGLT1 expression in colonocytes and increased luminal glucose concentrations. SGLT1 and DRA expression was ablated by either TcdA or TcdB during acute infection, but NHE3 was decreased in a TcdB-dependent manner. The localization of key proteins that link filamentous actin to the ion transporters in the apical plasma membrane was unchanged. However, *Sglt1, Nhe3*, and *Dra* were drastically reduced at the transcript level, implicating downregulation of ion transporters in the mechanism of diarrhea during CDI.

**Conclusions:**

CDI increases intestinal permeability and decreases apical abundance of NHE3, SGLT1, and DRA. This combination likely leads to dysfunctional water and solute absorption in the large bowel, causing osmotic diarrhea. These findings provide insights into the pathophysiological mechanisms underlying diarrhea and may open novel avenues for attenuating CDI-associated diarrhea.

## GRAPHICAL ABSTRACT

**Figure:**
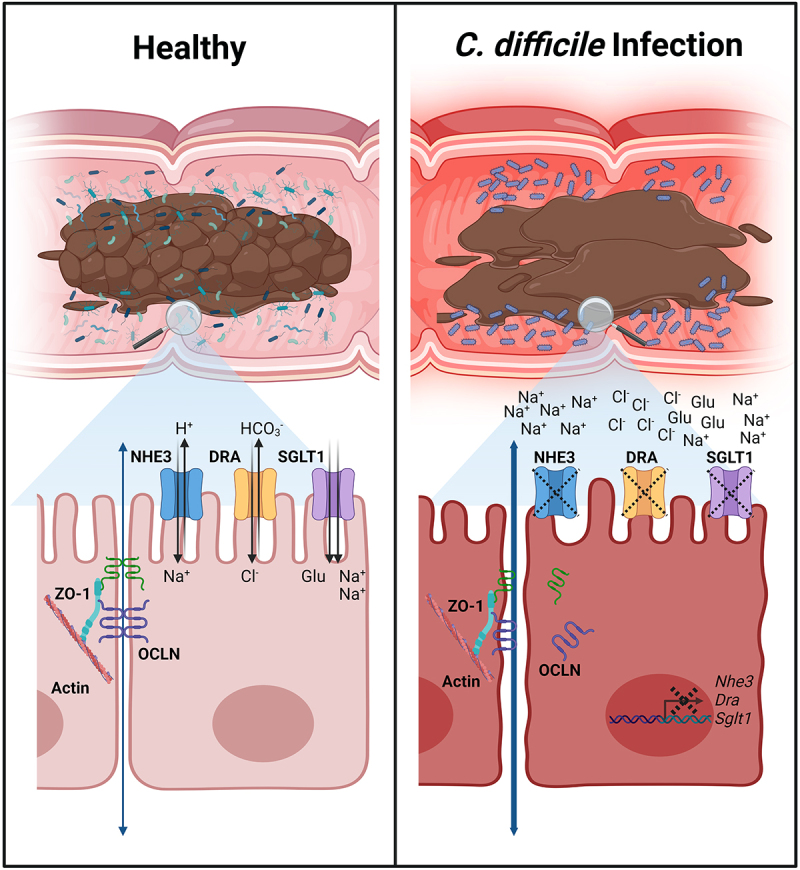

## Introduction

*Clostridioides difficile* infection (CDI) is the leading cause of hospital-acquired diarrhea in the USA, posing a significant burden on patients and the healthcare system.^1,2^ This antibiotic-associated infection causes ~ 250,000 cases and ~ 14,000 deaths in the USA each year, contributing $1.5 billion annually to the cost of healthcare.^3,4^ Furthermore, 25% of CDI patients experience a recurrent infection within two to 8 weeks of the initial infection, with each episode increasing subsequent risk of recurrence.^1^ *C. difficile* is transmitted via the fecal-oral route, where hardy spores traverse the gastrointestinal (GI) tract to germinate in the small intestine and colonize in the colon as vegetative cells.^5^ Infection results in symptoms ranging from mild to severe diarrhea, pseudomembranous colitis, and, in severe cases, toxic megacolon, sepsis, and death.^5^ Diarrhea provides a route of spore dispersal onto skin, clothes, and environmental surfaces that remain contaminated after symptom resolution.^6^ Environmental contamination is difficult to eliminate and perpetuates pathogen spread.^6^ Therefore, a better understanding of the mechanisms underlying diarrhea and its impact on CDI severity may help improve patient outcomes and mitigate pathogen spread.

Pathogenesis during CDI is mediated by two glucosylating protein toxins, TcdA and TcdB, and may be exacerbated by a third toxin, *C. difficile* transferase.^7^ Upon production in the colon, TcdA and TcdB bind host cell receptors and are endocytosed.^8^ Acidification of the endosome causes conformational changes in the toxins that lead to pore formation and delivery of the glucosyltransferase enzymes into the host cytosol.^8^ There, the enzymes irreversibly inactivate Rho-family GTPases via glucosylation, disrupting the host cytoskeleton and leading to the production of proinflammatory cytokines, ultimately resulting in cytopathy and cell death.^8^ Additionally, TcdB concentrations at and above 0.1 nM induce glucosyltransferase-independent epithelial injury through the production of reactive oxygen species that results in a necrotic cell death.^8^ Despite the understanding that TcdA and TcdB are the main determinants of disease, the role of each toxin during infection is not fully defined.^9,10^ A recent study from our lab reaffirmed that both TcdA and TcdB alone can cause symptoms in the mouse model of CDI, and suggested that the toxins synergize to worsen diarrhea symptoms and disease outcomes.^9^

Studies of purified *C. difficile* toxins in the 1980s demonstrated that injection of TcdA into rabbit ileal and colonic loops, or challenging *ex vivo* intestinal tissue with TcdA caused tissue damage, increased mucosal permeability, fluid accumulation in the lumen, and increased Cl^–^ secretion.^11–15^ A follow-up study demonstrated that TcdA, but not TcdB, significantly increased intestinal permeability in rabbit ileal loops, suggesting that TcdA-induced paracellular permeability was the major driver of diarrhea.^16^ The idea that toxin-mediated tissue damage increases paracellular water and solute efflux has been adopted as a likely mechanism of diarrhea in the *C. difficile* field. However, our understanding and appreciation of the complex pathophysiology that underlies diarrheal conditions has improved in the past two decades through the molecular identification of solute and water transporters.^17^ An *in vitro* study demonstrated that TcdB caused internalization of the Na^+^/H^+^ Exchanger 3 protein, NHE3 (a.k.a. SLC9A3).^18^ Depletion of NHE3 was confirmed in CDI patient biopsies, which was accompanied by elevated Na^+^ and alkaline levels in patient stool.^19^ A more recent study demonstrated that the Cl^–^/HCO_3_^–^ exchanger protein, DRA (Downregulated in Adenoma; a.k.a. SLC26A3), was depleted in CDI patient biopsies and during murine rectal instillation of purified TcdA.^20^ The coupled function of NHE3 and DRA is the primary route of electroneutral NaCl absorption in the lower GI tract, and functionally inactivating mutations in either gene can cause congenital diarrhea.^21–24^ Moreover, in models of inflammatory diarrhea, increased paracellular permeability alone is not sufficient to cause diarrhea; rather, increased permeability and impairment of NHE3 is necessary for solute malabsorption and diarrhea.^25^ These findings led us to hypothesize that *C. difficile* toxins induce concerted changes in paracellular permeability and solute absorption and secretion during infection that cause spore-dispersing diarrhea. In this study, we examined this hypothesis and tested the previously proposed models of TcdA-induced diarrhea, with the aim of defining the mechanistic etiology of diarrhea during acute CDI.

## Results

### Both TcdA and TcdB contribute to diarrhea severity during murine CDI

**Figure 1.**
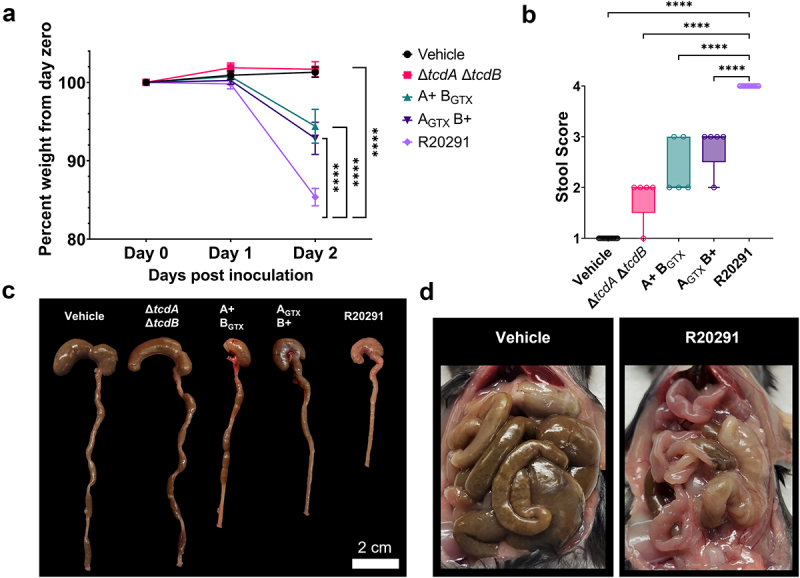
Diarrhea severity during C. difficile infection with R20291 is toxin-additive.

We previously observed that diarrhea was most severe in mice infected with the wildtype R20291 strain when compared to mutants that had mutationally impaired glucosyltransferase activity in either TcdA or TcdB (A+ B_GTX_ and A_GTX_ B+).^9^ To generate tissues for the experiments described below, we repeated previously published disease analyses and found consistent results.^9^ Weight loss was recorded and showed significant differences in percent weight loss at 2 days post-inoculation (dpi) between R20291 and A+ B_GTX_ , A_GTX_ B+, and Δ*tcdA*Δ*tcdB* (*p* <.0001) (Figure 1a). Mice inoculated with A+ B_GTX_ and A_GTX_ B+ strains lost significantly more weight than Δ*tcdA*Δ*tcdB*-inoculated mice (*p* <.0001; contrast not shown on graph), but there was no difference in weight loss between A+ B_GTX_ and A_GTX_ B+ (*p* = 0.8531; Figure 1a). Stool samples were scored by color and consistency at 2 dpi, demonstrating that R20291 consistently caused more severe diarrhea compared to toxin mutant strains (Figure 1b). These results are reflected by the empty ceca and colons from R20291-infected mice (Figure 1c,d). *In situ* visualization of the GI tract highlighted the empty and inflamed bowels observed during infection with wildtype *C. difficile* R20291 (Figure 1d). These data indicate that both TcdA and TcdB contribute to weight loss and diarrhea in our mouse model of CDI and provide the framework to examine the role of toxins in R20291-associated diarrhea during acute infection at 2 dpi.

(a) Percent weight change of mice inoculated with wildtype and mutant *C. difficile* strains demonstrates the toxin-additive nature of weight loss (*n =* 5-15 per group). (b) Stool scores are consistently more severe during R20291 infection compared to mutant strains (*n* = 5-15 per group). (c) Representative images of mouse ceca and colons during infection with wildtype and mutant strains at 2 dpi. Scale bar = 2 cm. (d) Abdominal cavities of representative R20291- or vehicle-inoculated mice at 2 dpi. Statistical significance was tested with two-way ANOVA with the Geisser-Greenhouse correction, followed by Tukey’s multiple comparisons test (A), or one-way ANOVA with unpaired Tukey’s t-test (b). *P < .05, ***P < .001, ****P < .0001, *P* > .05 was considered not significant.

### Intestinal permeability is increased through a size-selective paracellular pathway during *C.* *difficile* infection

**Figure 2.**
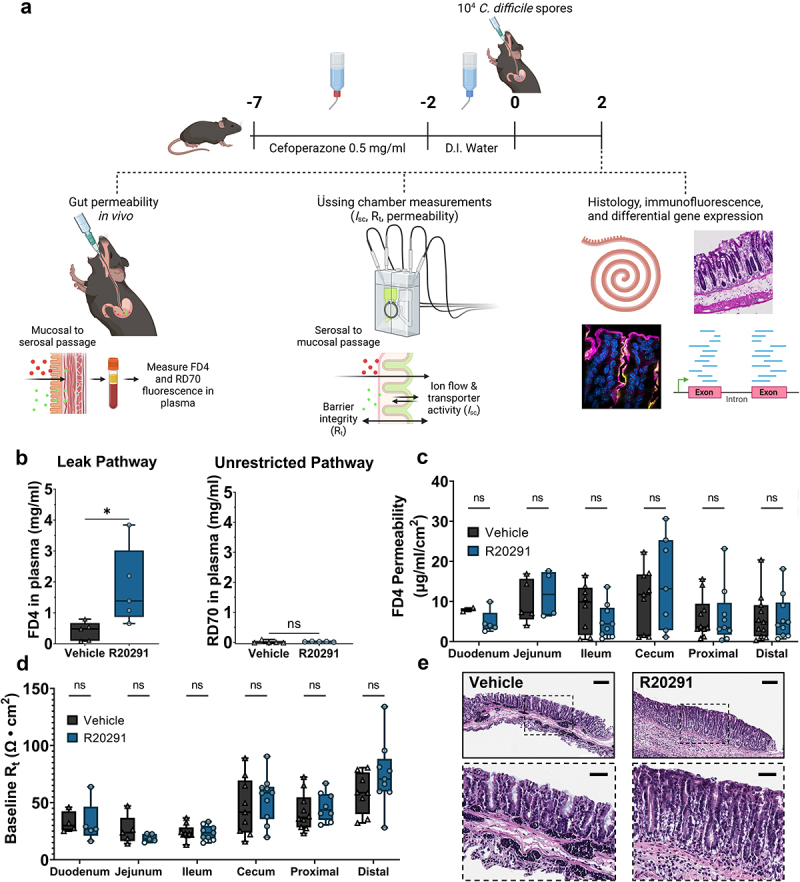
Intestinal permeability is increased *in* *vivo* via the leak pathway during infection.

To define the pathways involved in acute *C. difficile* diarrhea, we measured intestinal permeability in mice inoculated with the wildtype R20291 strain at 2 dpi by quantifying flux of orally gavaged FD4 and RD70 across the intestinal mucosa into the bloodstream (Figure 2a). Due to the extensive epithelial injury in the cecum and colon during CDI (Supplementary Figure S1), we expected to observe increased flux of both FD4 and RD70. Contrary to our expectations, increased intestinal permeability of FD4 (*p* = 0.038), but not RD70, was observed during acute CDI, implicating the size-selective leak pathway in this process (Figure 2b). We predicted that FD4 flux would be increased in *ex vivo* cecum and colon tissue, where extensive histopathological damage occurs (Supplementary Figure S1). Unexpectedly, there was no increase in permeability in large intestinal tissues, or anywhere in the intestinal tract of *C. difficile*-infected mice when tissue was measured in Üssing chambers (Figure 2c and Supplementary Figure S2). As an orthogonal measurement for barrier integrity, baseline transmucosal resistance (R_t_) was measured 60 min after mounting *ex vivo* tissue in Üssing chambers. There was no significant change in R_t_ between CDI and healthy mice across the entire intestinal tract (Figure 2d). After 60 min, these tissue segments were subjected to a course of ion transport inhibitors and stimulants to test electrogenic ion transport during CDI. Despite the addition of chemicals over the course of 120 min, there were no visible ischemic effects that would bias the barrier function and ion transport analyses (Figure 2e). Together, these results demonstrate that intestinal permeability is increased during CDI *in vivo*, potentially through the size-selective leak pathway. Barrier function measurements in Üssing chambers also showed that CDI caused no net change in R_t_ throughout the intestinal tract, despite the severe epithelial damage caused during infection.

(a) Graphical schematic of *C. difficile* infection model and analyses. (b) Paracellular flux is increased *in* *vivo* via the leak pathway during CDI, and there is no increase in permeability through the unrestricted pathway (*n* = 5 per group). (c) There is no difference in 4 kDa FITC-dextran (FD4) permeability between vehicle- and R20291-inoculated mice at 2 dpi across the intestinal tract when assessed in Üssing chambers (*n* = 2-11 per treatment per region). (c) Baseline transmucosal resistance (R_t_) in mucosa-submucosa preparations of the intestines is not significantly altered during CDI (*n* = 4-10 per treatment per region). (d) Representative H&E-stained mucosal preparations of distal colons after an Üssing chamber experiment. Scale bars = 100 μm. Statistical significance was tested with one-way ANOVA with unpaired Tukey’s t-test (b–d). *P < .05, *P* > .05 was considered not significant.

### *C. difficile* infection decreases Cl*^–^* secretory activity in the cecum and colon

**Figure 3.**
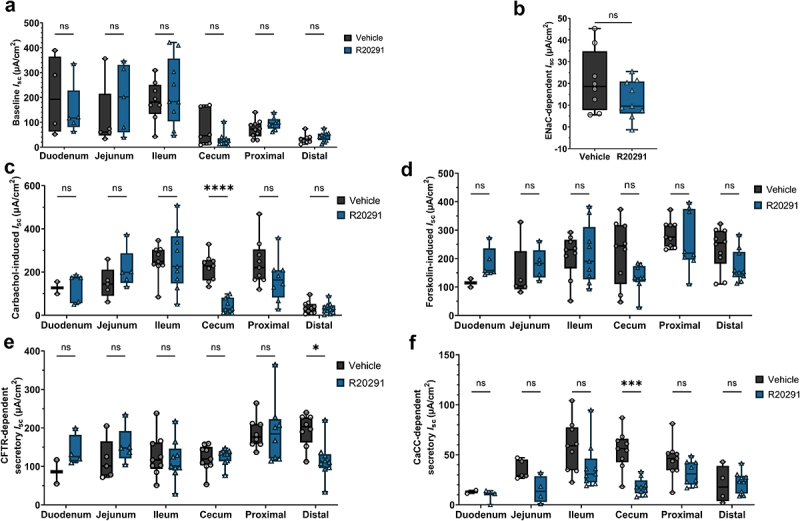
Chloride secretory channel activity is diminished in the cecum and distal colon during CDI.

The function of intestinal ion transporters during acute CDI was tested using electrophysiological measurements in Üssing chambers. The baseline short circuit current (*I*_sc_) was not significantly different between R20291-infected and control mice, suggesting a minimal effect of CDI on electrogenic ion transport (Figure 3a). The absorptive Epithelial Sodium Channel (ENaC), which is functionally relevant in the distal colon, was tested through inhibition with amiloride. There was no significant impairment of ENaC function during CDI (Figure 3b and Supplementary Figure S3). Cystic Fibrosis Transmembrane Conductance Regulator (CFTR) is responsible for most anion secretion in response to increased intracellular Ca^2+^ and cyclic dinucleotides, and Calcium activated Chloride Channels (CaCC) contribute to Cl^–^ secretion. To assess their function during CDI, the acetylcholine analogue, carbachol (Cch), and the adenylate cyclase-inducing chemical, forskolin (Fsk), were applied sequentially. The addition of Cch revealed that the secretory potential of the cecum was severely decreased during CDI (*p* < .0001), while there were no significant effects on other regions of the intestinal tract (Figures 3c,d). The CFTR-dependent portion of stimulated secretion was measured by adding a maximal dose of the inhibitor R-BPO-27. This demonstrated that CFTR function was significantly impaired in the distal colon during CDI (*p* < .05; Figure 3f). CaCC-dependent secretion was measured by the addition of the inhibitor CaCC-A01. CaCC activity was reduced nearly five-fold in the cecum during CDI (*p* < .001), accounting for the reduction in secretory potential after Cch stimulation (Figure 3f). These results suggest that neither Cl^–^ hypersecretion nor ENaC-mediated Na^+^ malabsorption are factors in diarrhea during CDI.

(a) Baseline short circuit current (*I*_sc_) is not significantly altered in the lower GI tract during CDI (*n* = 4-10 per treatment per region). (b) Function of the absorptive Epithelial Sodium Channel (ENaC) is unaltered in the distal colon during infection (*n* = 8-9 per group). (c) Addition of carbachol causes Ca^2+^-induced Cl^–^ secretion, revealing reduced Cl^–^ secretory potential in the cecum during CDI (*n* = 2–11 per group per region). (d) Forskolin-induced cAMP-dependent Cl^–^ secretion was unchanged during CDI (*n* = 2–10 per group per region). (e) Cystic Fibrosis Transmembrane Conductance Regulator (CFTR) activity was significantly decreased in the distal colon during infection (*n* = 2–9 per group per region). (F) Calcium-activated Chloride Channel (CaCC) activity is significantly impaired in the cecum during CDI (*n* = 2–9 per group per region). Statistical significance was tested with one-way ANOVA with unpaired Tukey’s t-test (A-F). *P < .05; ***P < .001; ****P < .0001, *P* > 0.05 was considered not significant.

### *C.* *difficile* infection causes loss of SGLT1 expression and function in the colon

**Figure 4.**
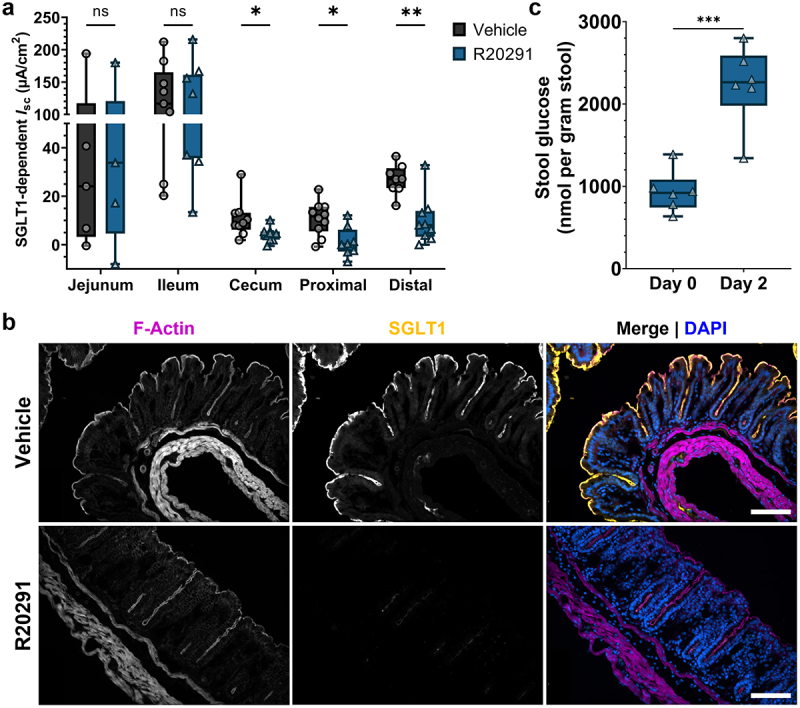
Colonic SGLT1 function is impaired, and expression diminished during CDI.

We determined the function of Sodium-dependent Glucose Transporter 1 (SGLT1; a.k.a. SLC5A1), which is critical for absorption of glucose/galactose, Na^+^, and water through the apical cell membrane in the small intestine.^26^ The function of SGLT1 in the presence of 11 mM glucose was tested through inhibition with phlorizin. There was no effect in SGLT1 activity in the jejunum or ileum, but unexpectedly, its function was significantly impaired in the cecum, proximal colon, and distal colon during CDI (*p <* .05) (Figure 4a). Using immunofluorescence microscopy, we found that SGLT1 expression was completely ablated in the colon during infection with R20291 (Figure 4b). SGLT1 is responsible for the majority of small intestinal glucose uptake, and the physiological relevance of SGLT1 in the colon is unknown. However, analysis of fecal samples revealed a greater than two-fold increase in stool glucose in R20291-infected mice at the same timepoint at which SGLT1 function and expression are decreased, suggesting that SGLT1 may be important for reabsorbing excess glucose in the colon (Figure 4c). The role of SGLT1 in carbohydrate, Na^+^, and water absorption is consistent with a model where loss of SGLT1 expression could contribute to diarrhea during CDI.^27^

(a) Sodium-dependent Glucose Transporter 1 (SGLT1) activity is reduced in the cecum, proximal colon, and distal colon during CDI (*n* = 5-10 per group per region). (b) Representative immunofluorescence images of the distal colon show a near complete ablation of SGLT1 expression at the apical surface during infection. Magnification is 20× and scale bars = 50 μm. (c) As a correlation to loss of colonic SGLT1, stool glucose concentration is increased two-fold during infection (*n* = 5 per group). Statistical significance was tested with a one-way ANOVA with unpaired Tukey’s t-test (A & C). * *P* < .05, ** *P* < .01, *P* > .05 was considered not significant.

### SGLT1 and DRA expression is decreased by either TcdA or TcdB, but decreased NHE3 expression is TcdB-dependent

**Figure 5.**
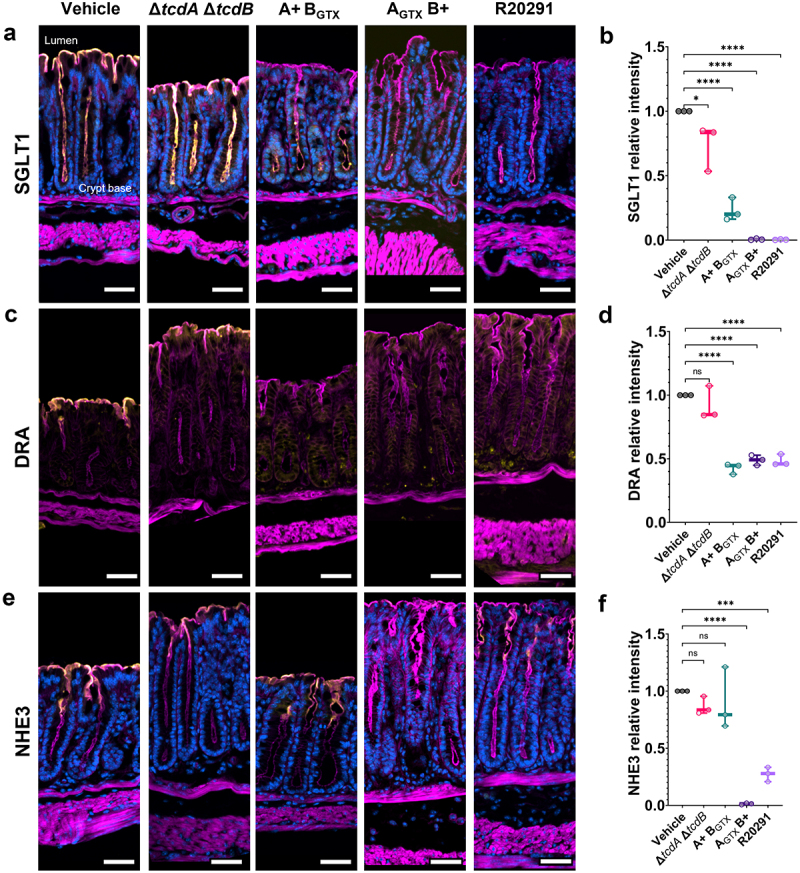
SGLT1 and DRA expression is decreased by TcdA or TcdB activity, but NHE3 depletion is TcdB-dependent during infection.

NHE3 and DRA are depleted during human CDI and are predicted factors in causing diarrhea.^18–20^ Given our observation that both TcdA and TcdB contribute to weight loss and diarrhea, we hypothesized that each toxin could have a different effect on the expression of ion transporters implicated in diarrhea. Using immunofluorescence microscopy, we found that SGLT1 expression was significantly decreased in the distal colon in response to either or both toxins but was largely present during colonization with a toxin-negative strain (Figures 5a, b and Supplementary Figure S4). Specifically, the expression of SGLT1 was ablated in the presence of functional TcdB, but in the A+ B_GTX_-infected mice, partial expression remained in the crypt base (Figure 5a).

(a,c,e) Immunofluorescence staining of ion transporters (gold), F-actin (magenta), and nuclei (blue) in the distal colon. (a & b) SGLT1 expression in the distal colon is decreased completely by TcdB, but low crypt expression remains in the presence of functional TcdA alone (*n* = 3 per group). (c,d) Distal colon DRA expression is decreased by either active TcdA or TcdB. (e,f) Distal colon NHE3 expression is only significantly reduced by functional TcdB (*n* = 3 per group). (a, c, e) Magnification is 20× and scale bars = 50 μm. Statistical significance was tested with a one-way ANOVA with unpaired Tukey’s t-test (b, d, f). *P < .05, ***P < .001, ****P < .0001, *P >* .05 was not considered significant.

DRA is functionally linked to NHE3 and is expressed in a low-to-high gradient from the proximal to distal colon in rodents.^28^ DRA has been shown to be downregulated at the protein but not mRNA level in mice intrarectally instilled with TcdA, but this has not been assessed during infection.^20^ Immunofluorescence analysis revealed that DRA expression was significantly decreased in the distal colon in response to functional TcdA or TcdB, with no additive outcome when both functional toxins were present (Figures 5c,d and Supplementary Figure S4).

**Figure 6.**
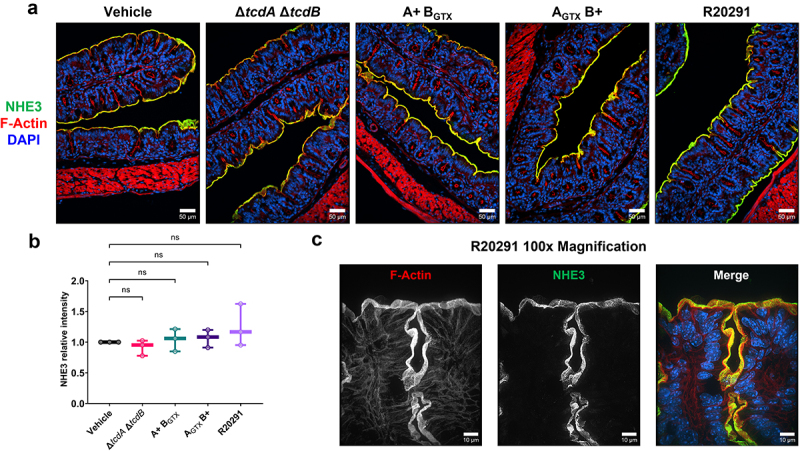
NHE3 expression in the proximal colon is unaltered during infection with wildtype or mutant *C.* *difficile* strains.

NHE3 is present in a gradient of high-to-low expression from the proximal to the distal colon in rodents.^28^ No studies have examined NHE3 expression in animal models of CDI. NHE3 expression was significantly decreased in a TcdB-dependent manner in the distal colon (Figure 5e,f and Supplementary Figure S4). Unexpectedly, NHE3 expression in the proximal colon, where it is more highly expressed, was unaffected during CDI (Figure 6a-c).

(a) Representative images show that apical NHE3 expression in the proximal colon is unchanged when inoculated with the vehicle control or toxigenic *C. difficile*. Magnification is 20× and scale bars = 50 μm. (b) The relative intensity of NHE3 show that there are no quantifiable changes in NHE3 expression in the proximal colon during CDI (*n* = 3 per group). (c) Higher magnification imaging (100×) show the apical staining patterns of NHE3 on proximal colon epithelial cells in a mouse infected with wildtype *C. difficile* (scale bars = 10 μm). Statistical significance was tested with one-way ANOVA with unpaired Tukey’s t-test (b). *P >* .05 was not considered significant.

**Figure 7.**
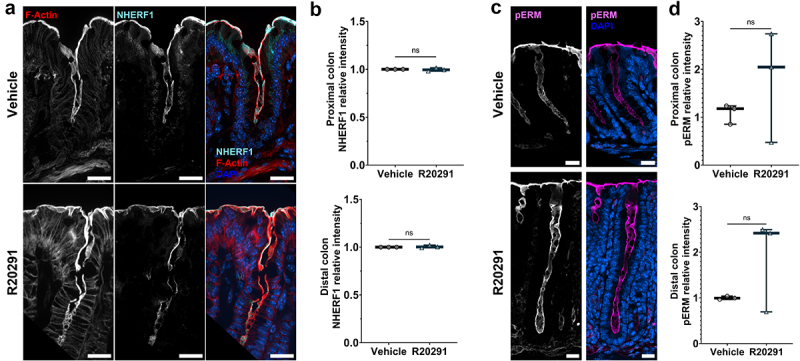
Expression of the cytoskeletal proteins NHERF1 and pERM is not altered in the colon during CDI.

NHE3 is internalized in response to TcdB glucosyltransferase activity *in vitro*.^18^ It was hypothesized that internalization is caused by TcdB-dependent deactivation of Rho-GTPases, which disrupts the cytoskeletal protein group Ezrin/Radixin/Moesin (ERM) that is linked to Na^+^-H^+^ Exchanger Regulatory Factor (NHERF) family proteins that anchor NHE3 to the cell membrane.^18,29^ We used immunofluorescence microscopy to quantify the expression and observed localization of NHERF1 and the active, phosphorylated ERM (pERM). There was no significant effect on NHERF1 or pERM expression and localization during infection with R20291, suggesting the potential for another mechanism of NHE3 depletion during infection (Figure 7a–d). Together, these data demonstrate the ability of both TcdA and TcdB to affect the expression of absorptive ion transporters in the distal colon, which likely contributes to the pathophysiology of diarrhea during CDI.

(a,b) Na^+^/H^+^ Exchanger Regulatory Factor 1 expression and localization is unaltered during infection (*n* = 3 per group per region). Magnification is 60× and scale bars = 20 µm. (c,d) Phosphorylated-Ezrin/Radaxin/Moesin (pERM) expression is not significantly altered in the colon during infection (*n* = 3 per group per region). Magnification is 40× and scale bars = 20 µm. Statistical significance was tested with one-way ANOVA with unpaired Tukey’s t-test (b & d). *P >* .05 was not considered significant.

### *Sglt1*, *Dra*, and *Nhe3* mRNA expression is downregulated in the colon in a toxin-specific manner

**Figure 8.**
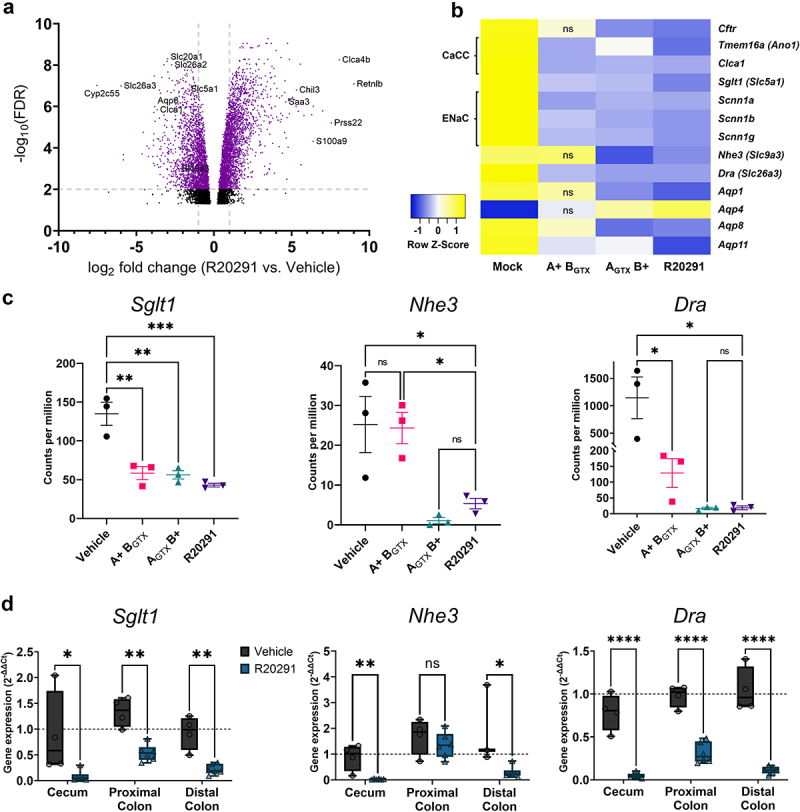
Transcriptomic analyses indicate that *Sglt1*, *Nhe3*, and *Dra* transcripts are downregulated in the distal colon during infection.

Since NHERF1 and pERM expression were unaltered during CDI, we hypothesized that NHE3 was decreased at the transcript level. We used RNA-sequencing as an unbiased approach to uncover toxin-dependent effects on mRNA levels. Transcriptomic analysis of the distal colon identified the potent upregulation of genes involved in the innate immune response including *Retnlb, Clca4b, Prss22, S100a9, Chil3*, and *Saa3* (Figure 8a and Supplementary Table S1). The most highly downregulated gene during infection was the cytochrome p450 family protein-encoding gene *Cyp2c55*, which has been previously reported.^10^ *Dra* (*Slc26a3*) was the second-most downregulated gene, followed by other solute and water transporters *Aqp8*, *Clca1*, *Slc20a1*, *Slc26a2*, *Nhe3* (*Slc9a3*), and *Sglt1* (*Slc5a1*).

(a) A volcano plot of differential expression data generated through RNA-sequencing of the distal colon during infection with R20291 or the vehicle control at 2 dpi (*n* = 3 per group) (b) Heatmap showing expression averages (*n* = 3 per group) of genes implicated in water and solute transport. High expression is shown in yellow and low in dark blue. (c) Gene expression of *Sglt1, Nhe3*, and *Dra* as measured by RNA-sequencing. Data are shown as counts per million, where the counts of each transcript were divided by the sum of library counts, then multiplied by one million. (d) Relative fold gene expression (2^−ΔΔCt^) of *Sglt1, Nhe3*, and *Dra* in isolated epithelial cells from healthy and infected tissues as measured by qRT-PCR (*n* = 3-6 per group). *Gapdh* C_t_ values were subtracted from the C_t_ of *Sglt1, Dra*, or *Nhe3* to generate ΔC_t_ values. ΔΔC_t_ values were calculated against a reference sample that consisted of cDNA from all mice in the vehicle control group. Statistical significance for RNA-sequencing data (a & b) was tested by normalizing transcriptomic data by trimmed mean of M-values, then performing differential expression analysis by estimating the mean-variance relationship of log_2_ counts per million of each transcript per samples in limma-voom and applying a False Discovery Rate cutoff of *P <* .05 for adjusted p-values. Statistical significance for expression comparisons between multiple strains (c) or two conditions (d) were tested with two-way ANOVA and multiple Tukey’s t-tests, or one-way ANOVA with unpaired Tukey’s t-test, respectively. *P < .05, **P < .01, ***P < .001, *P >* .05 was not considered significant.

Transcript expression comparisons between vehicle control mice and A+ B_GTX_, A_GTX_ B+, and R20291-inoculated mice were conducted to explore toxin-dependent effects on expression (Suplementary Tables S2 and S3). Consistent with protein expression data, there was a significant decrease in *Sglt1* and *Dra* expression in mice inoculated with mutant and wildtype strains (Figure 8b, c). Transcript data also supported TcdB-dependent downregulation of *Nhe3* (Figure 8b,c). Transcript expression changes in *Sglt1, Dra*, and *Nhe3* were confirmed in epithelial cells isolated from the cecum, proximal colon, and distal colon of control and wildtype *C. difficile* R20291-infected mice at 2 dpi using qRT-PCR. Relative gene expression analyses supported the downregulation of *Sglt1* and *Dra* from the cecum to distal colon, as well as downregulation of *Nhe3* in the cecum and distal colon, but not the proximal colon (Figure 8d). These data demonstrate that the absorptive ion transporters SGLT1, NHE3, and DRA are decreased at the transcript level during acute CDI.

We assessed transcript changes of ion transporters that were tested using Üssing chambers in the distal colon using the RNA-seq dataset. There were significant decreases in *Cftr* expression in mice inoculated with TcdB-positive strains only (Figure 8b), which may affect CFTR activity (Figure 3e). All three strains caused significant decreases in the putative CaCCs, *Tmem16a* (a.k.a. *Ano1*) and *Clca1*. There was also significant downregulation of the ENaC-encoding genes *Scnn1a, Scnn1b*, and *Scnn1g* (Figure 8b). Decreased transcript abundance of CaCC- and ENaC-encoding genes did not reflect the relatively stable function of these ion transporters in the distal colon during CDI (Figures 3b,f and 6b), highlighting the complexity of expression and function of ion transporters during disease states.

Finally, aquaporins 1, 4, 8, and 11 were among the most differentially expressed genes during CDI (Figure 8b). Transcript levels of aquaporins 1 and 4 were decreased or increased, respectively, and were dependent on TcdB activity (Figure 8b). The A+ B_GTX_ strain reduced the expression of *Aqp8* and *Aqp11*, but *Aqp8* downregulation was more potent in mice infected with strains that possess functional TcdB (Figure 8b). Decreased expression of *Aqp11* was most severe in R20291-infected mice, suggesting a synergistic effect in the presence of both functional toxins (Figure 8b). These data suggest that aquaporin proteins could also have a previously uncharacterized role in pathophysiology of water and solute transport during CDI.

## Discussion

Diarrhea during CDI can be mild and self-limiting, but in severe cases can cause dehydration through significant water and electrolyte loss. Additionally, diarrhea is the main source of pathogen spread between patients and onto environmental surfaces, which can be spore reservoirs for future outbreaks. Despite the importance of diarrhea during CDI, we do not fully understand the mechanisms underlying this symptom. Previous studies have used cell culture models, intrarectal instillation of toxins in mice, ileal loop injections in rabbits, and human patient samples to examine the effects of toxins and CDI on tight junctions and the expression of key ion transporters.^11,13,15,16,19,20^ These studies implicate increased paracellular leakage, Cl^–^ secretion, and/or expression loss of electroneutral ion exchangers NHE3 and DRA as potential causes of diarrhea. However, we are not aware of a paper that takes a comprehensive view of these mechanisms in the context of an *in vivo* infection. In this study, we sought to define the factors underlying diarrhea and to determine the effect of *C. difficile* toxins on this process using a physiologically relevant acute infection model.

Previous studies have tested the impact of toxins on permeability and Cl^–^ secretion using Üssing chambers.^11,15,30,31^ These studies used *ex vivo* rabbit ileal tissue that was exposed to crude or purified toxins in the chamber system over the course of several hours. The mouse infection model allows us to study permeability and electrogenic ion transport in a physiological disease setting that includes a fully functioning immune response. We tested permeability *in vivo* by gavage fasted mice with FD4 and RD70. Since *C. difficile* causes significant epithelial damage, we expected to see increased permeability of RD70 through the unrestricted pathway, similar to the dextran sodium sulfate model of colitis.^32^ Instead, this experiment revealed that permeability is increased through the leak pathway, shown by increased FD4 in plasma. Based on previous studies, this result suggests that intestinal permeability during CDI is increased through tight junction-dependent mechanisms.^32,33^ We tested tissue permeability in Üssing chambers to delineate where in the intestinal tract permeability was increased. Unexpectedly, there was no increase in tissue permeability in the duodenum, jejunum, ileum, cecum, proximal colon, or distal colon, despite the extensive pathological damage in the cecum and colon during CDI. The simplest explanation is that the small tissue size used for the Üssing chamber permeability analysis may not provide the sensitivity necessary to measure FD4 permeability. However, it is tempting to speculate that permeability may be increased through dynamic processes such as transmigration of polymorphonuclear immune cells through the epithelial layer, which would require circulating immune cells.^34^ An alternative explanation could be that *C. difficile* infection increases sampling of luminal antigens, resulting in increased FD4 in sera.

Maintaining ion transport is essential for gut homeostasis and solute absorption and secretion.^17^ There were no significant changes in baseline R_t_ or *I*_sc_ in Üssing chambered tissues during CDI. Previous studies have found that both tissue R_t_ and transepithelial resistance in cell cultures are decreased after acute exposure to *C. difficile* toxins.^14,35,36^ To the best of our knowledge, this is the first time that R_t_ has been evaluated in tissues during a physiological *C. difficile* infection. These data, along with permeability data, suggest that dynamic epithelial remodeling occurs in the lower GI during infection, which may not be active in homogenous cell culture and explant intoxication models. Indeed, studies have reported increased crypt length and epithelial cell differentiation during infection, which may serve, in part, to reinforce the mucosal barrier during CDI.^37,38^ Electrogenic ion transport measurements revealed no effects on ENaC function during infection. Contrary to previous findings,^14,15^ there was no increase in Cl^–^ secretion after addition of carbachol and forskolin to infected tissue. In fact, there was a decrease in Cl^–^ secretory potential in the cecum during CDI. CFTR activity was decreased significantly in the distal colon, and the loss of Cl^–^ secretion in the cecum was accounted for by reduced CaCC activity. Decreased CaCC and CFTR activity might be a result of the severe epithelial damage that occurs in the cecum and distal colon in the mouse CDI model. However, specific decreases in function of either CaCC in the cecum and CFTR in the distal colon may suggest the involvement of more physiological mechanisms of regulation during infection. Overall, these results strongly suggest that diarrhea during acute CDI is not a result of Cl^–^ hypersecretion, as exemplified by Cl^–^ rich cholera diarrhea, nor is it mediated by lack of Na^+^ absorption by ENaC, which is a pathophysiological factor of diarrhea during Salmonella-induced enteritis.^17,39,40^

Electrogenic measurements in Üssing chambers revealed that SGLT1 function was significantly impaired in the cecum and colon during CDI. Immunofluorescence imaging further suggested that this loss of function is due to a near complete ablation of SGLT1 expression. There was a two-fold increase in stool glucose during acute CDI compared to zero dpi. SGLT1-mediated carbohydrate absorption is thought to be mostly relevant in the small intestine and is only expressed at low levels in both rodent and human colons, thus, its function in the colon is incompletely understood.^41,42^ SGLT1 can actively uptake glucose in rat, pig, and human colons, albeit at a lower rate compared to the small intestine.^43,44^ This result is intriguing since *C. difficile* represses toxin production in the presence of glucose *in vitro* through a mechanism of carbon catabolite repression.^45,46^ Moreover, a high-carbohydrate diet rich in corn starch, casein, maltodextrin, and sucrose was protective against severe CDI.^47^ Finally, the two dpi timepoint is when mice undergo the most severe CDI symptoms, and they typically begin resolving by 4 dpi.^9^ It is therefore conceivable that inactivation of SGLT1 in the colon during severe CDI would increase luminal glucose, decrease toxin production, and thus promote disease resolution.

We previously observed that diarrhea and weight loss were increased during infection with the epidemic B1/NAP1/PCR-ribotype 027 R20291 strain, when compared to mutants with impaired glucosyltransferase activity of TcdA or TcdB.^9^ This led us to hypothesize that each toxin had unique effects on host physiology that synergize to cause severe diarrhea. We used immunofluorescence microscopy to quantify the relative abundance of SGLT1, NHE3, and DRA during acute infection. These experiments revealed that both TcdA and TcdB decrease DRA and SGLT1 expression significantly during infection. This finding is in contrast to a previous study, where DRA protein expression was reduced by intrarectal administration of TcdA, but not TcdB.^20^ The difference in our results are most likely due to the repeated exposure of host cells to TcdA and TcdB during the course of infection compared to 4 h of exposure to either toxin alone.^20^ An alternate explanation could be that TcdA decreases DRA expression in a glucosyltransferase-independent manner, which is why DRA abundance was decreased in mice infected with either A+ B_GTX_ and A_GTX_ B + .Congruent with a previous study,^18^ NHE3 expression was only decreased by strains that expressed functional TcdB. Unexpectedly, NHE3 expression was only decreased in the distal colon, where its expression is already very low, but not in the proximal colon. It has previously been shown that TcdB causes internalization of NHE3 in cell cultures.^18^ The authors hypothesized that glucosylation of Rho GTPases by TcdB could lead to disruption of ERM and NHERF proteins, leading to internalization of NHE3.^18^ We examined the expression of pERM and NHERF1 during infection at 2 dpi and found no quantifiable differences. However, this does not preclude the possibility of internalization by this pathway, rather, we may be capturing a snapshot of infection where NHE3 has already been internalized and the cytoskeleton has undergone rearrangements that do not include returning NHE3 to the apical cell surface.

We used an untargeted RNA-sequencing approach to interrogate dysregulation of ion transporters in the distal colon during infection. These data revealed a significant decrease in transcript levels of *Sglt1, Nhe3, and Dra* during infection. Downregulation of *Nhe3* at the transcript level is supported by a previous study, where *Nhe3* expression was decreased in human colonic organoids that were exposed to *C. difficile*.^19^ The decreased mRNA levels of *Dra* we observed during infection contradicts existing data from mice intrarectally instilled with TcdA or TcdB.^20^ Those mice were exposed to toxins for 4 h before mice were euthanized and gene expression analyses performed. Taken together, the data from these two studies suggest that the pathophysiological mechanisms leading to downregulation of *Dra* at the mRNA level likely require extended exposure to *C. difficile* toxins during infection. Finally, the untargeted RNA-sequencing approach also highlighted four aquaporin genes that were either up- or down-regulated during infection, which may play a role in water transport that contributes to diarrhea.

Downregulation of NHE3, DRA, and SGLT1 at the transcript level is likely a major factor contributing to diarrhea during CDI, since the absence of each of these transporters alone in humans can cause congenital Na^+^, Cl^–^, or glucose-galactose malabsorption diarrhea.^21,22,41^ This downregulation response could augment or conflict with the idea that toxin-mediated perturbation of the cytoskeleton may disrupt trafficking of ion transporters to the apical membrane, leading to loss of function.^18^ Rapid regulation of NHE3 and DRA relies on endocytic recycling and trafficking of these transporters to the apical membrane of epithelial cells.^48^ It is possible that toxins disrupt membrane trafficking by inactivating Rho GTPases, which leads to downstream signals that decrease expression of each transporter gene. During chronic inflammatory bowel diseases, *Nhe3, Dra*, and *Sglt1* expression is inhibited by circulating proinflammatory cytokines.^49–51^ The inflammatory response to acute *C. difficile* infection leads to high serum levels of proinflammatory cytokines including interleukin-1β, IL-6, IL-8, IL-17A, interferon-gamma, and tumor necrosis factor-alpha.^52^ It is, therefore, conceivable that toxin-mediated inflammatory responses during CDI cause widespread transcriptional downregulation of *Sglt1, Dra*, and *Nhe3* in the cecum and colon. Additionally, increased paracellular flux through the leak pathway is upregulated in response to T cell-mediated increases in TNFα and IFNγ.^33,53^ These findings lead us to hypothesize that host inflammatory responses during CDI may increase paracellular efflux through the leak pathway, and decrease solute absorption by NHE3, DRA, and SGLT1 in the colon, leading to coordinated dysfunction and malabsorptive diarrhea. Paracrine signaling events would amplify the impact of toxins beyond the cells that undergo direct intoxication. Further experiments are needed to define the mechanisms underlying the pathophysiological changes in solute and water transport during CDI.

In conclusion, these data demonstrate that both TcdA and TcdB are necessary in the R20291 strain to cause pathophysiological changes leading to diarrhea during CDI. Diarrhea associated with CDI is likely a multifactorial condition caused by increased intestinal permeability, and decreased water, Na^+^, Cl^–^, and carbohydrate absorption by SGLT1, NHE3, and DRA. Questions remain about the pathophysiological cues that dysregulate these transporters, which will be addressed in future studies.

## Materials & methods

### Animals and ethics statement

Nine-week-old female C57BL/6J mice purchased from The Jackson Laboratory were allowed to acclimate to the facility for a week before any procedures were performed. Mice were housed in specific pathogen-free conditions with 12-h cycles of light and dark and used at 9–11 weeks of age. Cages were changed every 2 weeks to ensure clean bedding, and unless otherwise mentioned, they had free access to food and water. All studies were approved by the Institutional Animal Care and Use Committee at Vanderbilt University Medical Center under protocol M1700185–01. All authors had access to the study data and have reviewed and approved the final manuscript.

### Clostridioides difficile culturing

Mutant and wildtype *C. difficile* strains (Table 1) were cultured at 37°C in supplemented brain-heart infusion medium (BHIS) in an anaerobic chamber (90% N_2_, 5% H_2_, 5% CO_2_). Strains were stored at ^–^80°C in 20% glycerol stocks for long-term use. Spore stocks were prepared as previously described and stored at 4°C until use.^9^

**Table 1.** Key resources.

REAGENT or RESOURCE	SUPPLIER	CONCENTRATION/DOSE	IDENTIFIER, CATALOG NO., OR CITATION
**Antibodies**			
Rabbit serum anti-Dra	Dudeja Lab	1:100	^54,55^
Rabbit monoclonal anti-pERM (3726S)	Cell Signaling	1:200	AB_10560513
Rabbit polyclonal anti-Nhe3 (NBP1–82574)	Novus Biologicals	1:100	AB_11038394,^56^
Rabbit polyclonal anti-NHERF-1 (ab3452)	Abcam	1:100	AB_303814
Rabbit polyclonal anti-Sglt1 (576–610)	Kaji Lab	0.5 µg/ml	^57^
Goat anti-rabbit Alexa Fluor 488 (A-11008)	Invitrogen	1:1000	AB_143165
Goat anti-rat Alexa Fluor 568 (A-11077)	Invitrogen	1:1000	AB_2534121
**Bacterial Strains**			
*C. difficile* R20291	Minton Lab	10^4^ CFU	^9,58^
R20291 A_GTX_ B+	Kuehne Lab	10^4^ CFU	^9^
R20291 A+ B_GTX_	Kuehne Lab	10^4^ CFU	^9^
R20291 Δ*tcdA* Δ*tcdB*	Minton Lab	10^4^ CFU	^9,59^
**Chemicals, Reagents, and Other Materials**			
Alex Fluor 647 Phalloidin	Invitrogen	1:200	A22287
DAPI (4’,6-Diamidino-2-Phenylindole, Dihydrochloride)	Invitrogen	1 µg/ml	D1306
Fluorescein isothiocyanate-4 kDa dextran	Sigma Aldrich	80 mg/ml	46944
Rhodamine B isothiocyanate-70 kDa dextran	Sigma Aldrich	40 mg/ml	R9379
Amiloride hydrochloride	Sigma	10 µM	BP008
Phlorizin dihydrate	Sigma	0.1 mM	274313
Indomethacin	Sigma	10 µM	I7378
Carbachol	Sigma	10 µM	PHR1511
Forskolin	Sigma	10 µM	F3917
(R)-BPO-27	MedChemExpress	10 µM	HY-19778
CaCC-A01	Sigma	100 µM	208293
Spin-X UF 500 µL Centrifugal Concentrator, 10 kDa MWCO Membrane	Corning		431478
Micro sample tube Citrate 3.1%, 1.3 mL screw cap	Sarstedt		41.1350.105
Brain Heart Infusion Broth	Research Products International		B11000
Cefoperazone sodium salt	Sigma Aldrich	0.5 mg/ml	C4292
Taurocholic acid sodium salt hydrate	Sigma Aldrich		T4009
D-Cycloserine	Sigma Aldrich		C6880
Cefoxitin sodium salt	Sigma Aldrich		C4786
RNAlater Stabilization Solution	Invitrogen		AM7020
Zirconia-silica beads, 1 mm diameter	BioSpec		11079110Z
TaqMan Fast Advanced Master Mix	Thermo Fisher		4444557
Mm00445313_m1 *Slc26a3* Gene Expression Assay (FAM-MGB)	Thermo Fisher		N/A
Mm01352473_m1 *Slc9a3* Gene Expression Assay (FAM-MGB)	Thermo Fisher		N/A
Mm00451203_m1 *Slc5a1* Gene Expression Assay (FAM-MGB)	Thermo Fisher		N/A
Mm99999915_g1 *Gapdh* Gene Expression Assay (FAM-MGB)	Thermo Fisher		N/A
**Commercial Kits**			
Glucose Assay Kit	Abcam		ab65333
TURBO DNA-free kit	Invitrogen		AM1907
Monarch RNA cleanup kit (50 µg)	NEB		T2040
NEBNext rRNA depletion kit v2 (human/mouse/rat)	NEB		E7400
NEBNext Ultra II Directional RNA Library Prep Kit for Illumina	NEB		E7760
NEBNext Multiplex Oligos for Illumina (96 Unique Dual Index Primer Pairs Set 3)	NEB		E6444
QIAShredder	Qiagen		79656
RNeasy Plus Mini Kit	Qiagen		74134
QuantiTect Reverse Transcription Kit	Qiagen		205311
**Experimental Models: Organisms**			
Mice: Female C57BL/6J	The Jackson Laboratory		000664
**Software**			
FIJI	National Institutes of Health		
GraphPad Prism	GraphPad Software Inc.		
Microsoft Excel	Microsoft		

### *Clostridioides difficile* infection

Ten-week-old C57BL/6J mice were treated with cefoperazone (0.5 mg/ml) antibiotics in drinking water *ad libitum* for 5 days, then returned to normal drinking water 2 days before inoculation. Mice were inoculated with 10^5^ CFU/ml spores suspended in 100 µl sterile PBS by transoral gastric gavage. Spore stocks and diluted inoculum from mutant and wildtype *C. difficile* strains were serially diluted onto BHIS + taurocholic acid (TA; 10% w/v) media to enumerate concentration before and after inoculation.

Mice were monitored daily, and weight and symptom severity were recorded. Stool samples were collected daily by scruffing mice and catching fresh stool in sterile, pre-weighed 1.5 ml microcentrifuge tubes. Stool samples were scored using a modified Bristol Stool Chart by color and consistency. In this scheme 1 = normal stool; 2 = well-formed, discolored stool; 3 = moist, soft, and discolored stool; 4 = wet tail, watery diarrhea, and empty bowels.^9^ Stool was then weighed, homogenized in PBS, and serially diluted on TCCFA media (TA 10% w/v; D-cycloserine 10 mg/ml; cefoxitin 10 mg/ml; fructose) to quantify *C. difficile* burden. At the experimental endpoint, mice were humanely euthanized by CO_2_ followed by cervical dislocation. The entire intestinal tract was excised and imaged. Tissues were processed as described below based on the experiment performed.

### *In vivo* paracellular permeability measurement

Mice were pre-treated with antibiotics then inoculated with wildtype *C. difficile* R20291, A+ B_GTX_, A_GTX_ B+, A_GTX_ B_GTX_, or PBS as described above. Paracellular permeability was measured using an established method.^32^ Two days post-inoculation (dpi), mice were placed into a new cage with water, but without food or bedding for 3 h to clear gut contents. A probe solution containing 80 mg/ml 4 kDa fluorescein isothiocyanate (FITC) dextran (FD4) and 40 mg/ml 70 kDa rhodamine B dextran (RD70) was prepared in sterile milliQ water and filter sterilized through a 0.2 µm filter into a new sterile tube. After 3 h of fasting, 250 µl of probe solution was orally gavaged into each mouse, while noting the exact time of gavage. One mock-inoculated mouse was orally gavaged with water for a plasma autofluorescence control. Mice were returned to their food- and bedding-less cage and monitored. Food and bedding were returned 90 min post-gavage. Three hours post-gavage, mice were euthanized, and blood was collected via heart stick using a 28 G insulin syringe. Equal volumes of blood were placed into 1.3 ml collection tubes containing 3.2% sodium citrate (Sarstedt). Tubes were gently mixed by inversion to prevent coagulation. After all the samples were collected, tubes were centrifuged at 1500 × g for 10 min at room temperature, and plasma was transferred to new 1.5 ml microcentrifuge tubes. A standard curve for FD4 and RD70 was prepared in duplicate, and plasma FITC and rhodamine B fluorescence was measured in technical duplicate on a BioTek Cytation 5 plate reader, and probe concentration in plasma was calculated exactly as described.^32^

### *Ex vivo* paracellular permeability measurement in Üssing chambers

To delineate the permeability of functionally distinct portions of the GI tract, mucosal-submucosal preparations of the duodenum, jejunum, ileum, cecum, proximal colon, and distal colon were assessed from R20291-infected and vehicle control mice at 2 dpi. Mice were euthanized, and tissues were excised by carefully cutting mesentery and connective tissue to prevent damage to the mucosa. Functional regions were delineated by sectioning the intestines and colon in equal thirds. Tissue for duodenum preparations was taken from the latter third of the duodenal segment to avoid Brunner’s glands and the pancreatic duct. Excised tissues were flushed with ice-cold, oxygenated Krebs-Ringer Buffer (KRB; 117 mM NaCl, 25 mM NaHCO_3_, 11 mM glucose, 4.7 mM KCl, 1.2 mM MgCl_2_, 2.5 mM CaCl_2_, and 1.2 mM NaH_2_PO_4_, pH 7.4). The segments were opened along the mesenteric border, and the seromuscular layer was removed in cold KRB using fine forceps under a dissecting microscope. Preparations were made from each segment, mounted in 0.1 cm^2^ sliders, and placed in Üssing chambers (Physiologic Instruments). The luminal and serosal surfaces of the jejunum, ileum, cecum, proximal colon, and distal colon were bathed in 4 ml KRB, bubbled with carbogen (95% O_2_, 5% CO_2_), and maintained at 37°C using a water-recirculating heating apparatus. The serosal surface of the duodenum was bathed in 4 ml KRB, and the luminal surface was bathed in HCO_3_^−^ free HEPES buffer (117 mM NaCl, 4.7 mM KCl, 2.5 mM CaCl_2_ , 1.2 mM MgCl_2_, 1.2 mM NaH_2_PO_4_, 24 mM sodium gluconate, 10 mM HEPES, and 11 mM glucose, pH 7.4), bubbled with 100% O_2_ to prevent CO_2_-stimulated HCO_3_^–^ secretion. To prevent prostaglandin synthesis, indomethacin (10 µM) was added immediately post-mounting to all serosal baths.

Mucosa-submucosa tissue preparations were stabilized for 15 min before administration of FD4 (0.25 mM) and RD70 (17.5 mM) to the serosal bath to measure permeability.^32^ The mucosal bathing solution was sampled in technical duplicate at 0 and 60 min after FD4 and RD70 addition, and the bath volume was maintained by adding fresh KRB. Fluorescence intensity of each 100 µl sample and standards were analyzed at excitation/emission of 495/525 nm for FD4 and 555/585 nm for RD70 on a BioTek Cytation 5 plate reader.^32,57^ Paracellular permeability was determined as the concentration of FD4 and RD70 that passed through the mucosal-submucosal preparations from serosal to mucosal baths as µg/ml/cm^2^.

### Ion transporter functional assessment in Üssing chambers

To assess function of electrogenic ion transporters, short-circuit current (*I*_sc_), tissue conductance (G_t_, mS/cm^2^), and transmucosal resistance (R_t_, Ω · cm^2^) were recorded under voltage-clamp conditions. G_t_ was measured every 5 sec with a bipolar pulse of ± 3 mV for 20 mS, and R_t_ was calculated as the inverse of G_t_. Baseline *I*_sc_ and R_t_ were recorded after the fluorescent dextran permeability measurements and before inhibitors were applied.

SGLT1 activity in the presence of 11 mM glucose was determined as 0.1 mM phlorizin sensitive *I*_SC_. ENaC activity was determined as 10 µM amiloride-sensitive *I*_SC_. Carbachol (10 µM) and forskolin (10 µM) were sequentially applied to the serosal bath to compare Ca^2+^ and cAMP-dependent Cl^–^ secretory responses. After the addition of forskolin, the contribution of CFTR and CaCC to stimulated Cl^–^ secretion was measured by administering the CFTR inhibitor (R)-BPO-27 (10 µM) followed by CaCC-A01 (100 µM).

After the Üssing protocol, mounted tissues were fixed in 10% Neutral Buffered Formalin (NBF) overnight, then embedded in paraffin by the Vanderbilt Tissue Pathology Shared Resource (TPSR). Sections were mounted onto slides and stained with H&E to validate the removal of the seromuscular layer and the lack of ischemic cell death during the experiment. H&E-stained slides were imaged at 40× magnification using a Leica SCN400 at the Digital Histology Shared Resource.

### Immunofluorescence staining, imaging, and analysis

Mice were euthanized two days post-inoculation, then the colon of each animal was excised. Colons were gently flushed with cold sterile PBS, then cut transversally and fixed in 2% paraformaldehyde (PFA) at room temperature for 2 h or 10% NBF at room temperature for 16 h. After 2 h, PFA-fixed tissues were washed three times in cold sterile PBS, then placed in a cold solution of 30% sucrose, 1% NaN_3_ and incubated for 16 h at 4°C. Colons were Swiss rolled and embedded in Optimal Cutting Temperature medium (OCT) in dry ice-cooled ethanol and stored at ^–^80°C until use. NBF-fixed tissues were rinsed three times in cold sterile PBS, then Swiss rolled and embedded in paraffin wax by TPSR.

Formalin-fixed paraffin embedded (FFPE) blocks were sectioned (7 µm) on a Microm HM 335E microtome, then dried overnight and kept at room temperature until use. Paraffin wax was removed using xylenes, then tissues were rehydrated in an ethanol gradient. Antigen retrieval was performed for 15 min using 0.1 M Citrate Buffer pH 6.0. Frozen colon blocks were sectioned (7 µm) on a Leica CM1950 Cryostat and kept at −80°C until use. OCT was removed by incubating in three sequential PBS washes. Both OCT and FFPE tissues were blocked for an hour at room temperature in a solution of PBS pH 7.4, 2% normal goat or donkey serum, and 0.3% Triton X-100.

Afterwards, the blocking solution was removed and replaced with the primary antibody diluted at a specified concentration (Table 1) in an antibody dilution buffer comprising PBS, 1% bovine serum albumin (BSA), and 0.3% Triton X-100. Slides were incubated with the primary antibody overnight (~16 h) at 4°C. The following day, slides were washed in three changes of PBS, then incubated for 1 h at room temperature in antibody dilution buffer containing secondary antibodies and/or phalloidin (Table 1). After an hour, slides were washed in three changes of PBS, incubated with DAPI for 1 min, then washed in PBS. Slides were cover slipped with ProLong Gold and allowed to cure overnight before sealing with clear nail polish and placing at 4°C in the dark until imaging.

Sections were imaged using a Nikon Spinning Disk confocal microscope equipped with a Yokogawa CSU-X1 spinning disk head, a Photometrics Prime 95B sCMOS monochrome camera, and 405 nm, 488 nm, 561 nm, and 647 nm diode laser lines. For each colon samples, at least three distinct regions of the proximal colon (first 1/3^rd^ of colon) and distal colon (last 1/3^rd^ of colon) were imaged for analysis in at least three mice per treatment. These images were acquired at 20× with a Plan Apo Lambda 20 × 0.75 NA WD 1.00 mm objective lens. Magnified images were acquired at 40×, 60×, or 100× with Pan Fluor 40× Oil DIC H N2 1.30 NA WD 0.20 mm, Plan Apo Lambda Oil 60 × 1.40 NA WD 0.13 mm, and Apo TIRF Oil 100 × 1.49 NA WD 0.12 mm objective lenses, respectively.

Relative fluorescence intensity was quantified as a metric for protein abundance using FIJI software (National Institutes of Health). A baseline threshold was generated with vehicle control images using Otsu’s method,^60^ and fluorescence intensity by area was calculated. The same threshold settings were applied across all treatments and regions of the colon for each protein analyzed. Fluorescence intensity data were averaged between different fields of view from the same region of the same mouse, and relative fluorescence intensity was calculated with reference to the vehicle control.

### Histopathological scoring

Pathological scoring of mouse intestinal tissues was performed as previously described.^9^ Briefly, vehicle control and R20291-infected mice were euthanized at 2 dpi, then the entire intestinal tract was excised. Functional regions of the intestines were separated as described above. Each segment was flushed with cold PBS, then opened longitudinally along the mesenteric border, laid flat between two pieces of Whatman filter paper, and fixed overnight at room temperature in 10% NBF. Tissues were washed three times in PBS, then each segment was Swiss rolled and placed in a tissue cassette in 70% ethanol until paraffin embedding. Tissues were cut to 7 µm sections on a Microm HM 335E microtome, then dried overnight and kept at room temperature until use. Paraffin wax was removed using xylenes, then tissues were rehydrated in an ethanol gradient. Sections were stained with hematoxylin & eosin (H&E; Vector Labs), and slides were scored by a board-certified gastrointestinal pathologist blinded to the conditions. Histological scoring was assessed for edema, inflammation, and epithelial injury based on previously published criteria.^9,61^ Images were acquired using a 10× objective on a BioTek Cytation 5 plate reader.

### Stool glucose measurement

Stool was collected into sterile, pre-weighed microcentrifuge tubes from mice inoculated with *C. difficile* R20291 (*n =* 5 per treatment) at day 0, prior to inoculation, and two-days post-inoculation. Tubes containing stool were weighed, and glucose was measured using the Glucose Assay Kit (Abcam) according to manufacturer’s instructions. Briefly, stool was mechanically homogenized in equal volumes of ice-cold assay buffer, then centrifuged at 10,000 × g for 5 min at 4°C. The supernatant was transferred to Spin-X UF 10 kDa molecular weight cutoff columns (Corning) and centrifuged at 10,000 × g for 10 min at 4°C to deproteinate the samples. Flowthrough was then used for colorimetric quantification of glucose concentration compared to standards provided by the manufacturer.

### Transcriptome Profiling of the distal colon

Mice inoculated with wildtype and mutant *C. difficile* strains, or the vehicle control were euthanized at 2 dpi, and a 0.5 cm segment of the distal colon was harvested and placed in RNAlater solution on ice. Tissues were washed with sterile PBS then transferred to fresh tubes containing 1 ml of TRIzol and 1 mm diameter Zirconia-silica beads. Colon samples were homogenized in an Omni Bead Ruptor 4 homogenizer for one minute in two 30 s intervals, then placed on ice for 5 min. Phases were separated by the addition of chloroform and centrifugation, then total RNA was precipitated, washed, and solubilized in nuclease-free water. DNA was depleted using TURBO DNA-free (Invitrogen), then RNA was purified using the Monarch RNA Cleanup Kit (NEB). Quantity and quality were assessed by spectroscopy.

RNA libraries were prepared by depleting eukaryotic ribosomal RNA with the NEBNext rRNA Depletion Kit prior to library synthesis with the NEBNext Ultra II Directional RNA Library Prep Kit and addition of multiplex oligos using the Unique Dual Index Primer Pairs Set 3 (NEB). Library quality control was performed using Qubit and Bioanalyzer 2100. Paired end 150 bp sequencing was performed on an Illumina NovaSeq 6000, with the aim of generating 50 million reads per sample. Sequencing data quality was assessed with FASTQC, then reads were aligned to the GRCm39 mouse genome (GenBank GCA_000001635.9) using the STAR alignment tool with default settings. Normalization by trimmed mean of M-values and differential expression was performed with limma-voom. Plots of differentially expressed genes were generated using GraphPad Prism or Heatmapper. Raw and analyzed data have been deposited on NCBI’s Gene Expression Omnibus and are accessible through GEO Series accession number GSE216919.

### Epithelial cell isolation and relative gene expression analyses

Epithelial cells were isolated from the cecum and colon of vehicle control and R20291-infected mice at 2 dpi based on an established protocol.^62^ Briefly, the lower intestine was excised, and luminal contents were gently flushed with cold PBS. The regions of interest were slid onto a metal gavage needle attached to a 5 ml syringe filled with air. A knot was affixed on the tissue segment nearest the gavage needle bulb with sterile 3–0 vicryl suture, and the tissue was inverted by sliding the intestine over the knot. Another suture knot was placed on the open end of the tissue, and then it was submerged in 4 ml Cell Recovery Solution (Corning) on ice. The tissue was inflated and deflated every 5 min for 30 min. After 30 min, the tissues were removed from solution and sterile surgical forceps were used to gently remove epithelial sheets from the tissue. RNA was isolated using QIAShredder lysis tubes and the Qiagen RNeasy Plus Mini Kit.

Relative gene expression of *Nhe3, Dra*, and *Sglt1* was measured using TaqMan-based quantitative Real Time-Polymerase Chain Reaction (qRT-PCR; Table 1). One microgram of RNA from each sample was synthesized to cDNA using the Qiagen QuantiTect Reverse Transcription Kit. cDNA was diluted 1:5 with nuclease-free water. The expression of each gene (as cycle threshold; C_t_) was measured within an intestinal region relative to the expression of *Gapdh* (ΔC_t_). ΔC_t_ values were compared to the expression of each transporter gene compared to *Gapdh* expression in a reference pooled sample comprising cDNA from vehicle control mice for each intestinal region. All qRT-PCR experiments were performed on a technical duplicate on a QuantStudio 6 Flex Real-Time PCR System.

### Statistical analyses

The data were plotted with GraphPad Prism, and statistical comparisons were determined using one-way ANOVA and unpaired Student’s t-test for two-group comparisons. For more than three groups, a two-way ANOVA, and Tukey’s multiple comparisons test were performed. *P* < .05 was considered to be the minimal significant difference.

## Supplementary Material

Supplemental Material

## Data Availability

Large datasets (RNA-seq) are available online on Gene Expression Omnibus with identifier GSE216919. Reagents used in this study are either commercially available or available upon request.

## Abbreviations

CDI: *Clostridioides difficile* infection
GI: gastrointestinal
NHE3: Na^+^/H^+^ Exchanger 3
DRA: Downregulated in Adenoma
BHIS: brain-heart infusion-supplemented medium
TA: taurocholic acid
TCCFA: taurocholic acid, D-cycloserine, cefoxitin and fructose agar
FITC: fluorescein isothiocyanate
FD4: 4 kDa FITC dextran
RD70: 70 kDa rhodamine B dextran
KRB: Krebs-Ringer Buffer
*I*_sc_: short-circuit current
R_t_: transmucosal resistance
G_t_: tissue conductance
SGLT1: sodium-dependent glucose cotransporter 1
ENaC: epithelial sodium channel
CFTR: cystic fibrosis transmembrane conductance regulator
PFA: paraformaldehyde
NBF: neutral buffered formalin
FFPE: formalin-fixed paraffin embedded
OCT: optimal cutting temperature embedding medium
ERM: Ezrin/Radixin/Moesin
NHERF: Na^+^/H^+^ exchanger regulatory factor
BI/NAP1/PCR-RT: restriction endonuclease analysis type B1, North American pulse-field gel electrophoresis type 1, polymerase chain reaction ribotype
qRT-PCR: quantitative real time-polymerase chain reaction

## BioRxiv preprint

https://doi.org/10.1101/2022.11.12.516162.

## Synopsis

*Clostridioides difficile* infection (CDI) causes increased intestinal fluid leakage and decreases the colon’s ability to absorb water and electrolytes. Using a mouse model of CDI, we found that intestinal permeability is increased through a size-selective pathway, and key absorptive ion transporters *Sglt1*, *Nhe3*, and *Dra* are downregulated. These factors likely cause diarrhea associated with CDI.

## Transcript profiling

RNA-seq data were uploaded into GEO database (GSE216919).

## References

[1] Lessa FC, Mu Y, Bamberg WM, Beldavs ZG, Dumyati GK, Dunn JR, Farley MM, Holzbauer SM, Meek JI, Phipps EC, et al. Burden of *Clostridium difficile* infection in the United States. N Engl J Med. 2015;372(9):825–23. doi:10.1056/NEJMoa1408913. 25714160 PMC10966662

[2] CDC. Antibiotic resistance threats in teh United States. Atlanta, GA: U.S. Department of Health and Human Services, CDC; 2019. doi:10.15620/cdc:82532.

[3] Guh AY, Mu Y, Winston LG, Johnston H, Olson D, Farley MM, Wilson LE, Holzbauer SM, Phipps EC, Dumyati GK, et al. Trends in U.S. burden of *Clostridioides difficile* infection and outcomes. N Engl J Med. 2020;382(14):1320–1330. doi:10.1056/NEJMoa1910215. 32242357 PMC7861882

[4] Zimlichman E, Henderson D, Tamir O, Franz C, Song P, Yamin CK, Keohane C, Denham CR, Bates DW. Health care–associated infections: a meta-analysis of costs and financial impact on the US health care system. JAMA Intern Med. 2013;173(22):2039–2046. doi:10.1001/jamainternmed.2013.9763. 23999949

[5] Smits WK, Lyras D, Lacy DB, Wilcox MH, Kuijper EJ. *Clostridium difficile* infection. Nat Rev Dis Primer. 2016;2(1):1–20. doi:10.1038/nrdp.2016.20. PMC5453186 27158839

[6] Bobulsky GS, Al-Nassir WN, Riggs MM, Sethi AK, Donskey CJ. *Clostridium difficile* skin contamination in patients with *C. difficile*-associated disease. Clin Infect Dis Off Publ Infect Dis Soc Am. 2008;46(3):447–450. doi:10.1086/525267. 18181742

[7] Kordus SL, Thomas AK, Lacy DB. *Clostridioides difficile* toxins: mechanisms of action and antitoxin therapeutics. Nat Rev Microbiol. 2022;20(5):285–298. doi:10.1038/s41579-021-00660-2. 34837014 PMC9018519

[8] Chandrasekaran R, Lacy DB. The role of toxins in *Clostridium difficile* infection. FEMS Microbiol Rev. 2017;41(6):723–750. doi:10.1093/femsre/fux048. 29048477 PMC5812492

[9] Peritore-Galve FC, Shupe JA, Cave RJ, Childress KO, Washington MK, Kuehne SA, Lacy DB. Glucosyltransferase-dependent and independent effects of *Clostridioides difficile* toxins during infection. PLoS Pathog. 2022;18(2):e1010323. doi:10.1371/journal.ppat.1010323. 35176123 PMC8890742

[10] Carter GP, Chakravorty A, Nguyen TAP, Mileto S, Schreiber F, Li L, Howarth P, Clare S, Cunningham B, Sambol SP, et al. Defining the roles of TcdA and TcdB in localized gastrointestinal disease, systemic organ damage, and the host response during *Clostridium difficile* infections. mBio. 2015;6(3):e00551. doi:10.1128/mBio.00551-15. 26037121 PMC4453007

[11] Mitchell TJ, Ketley JM, Haslam SC, Stephen J, Burdon DW, Candy DC, Daniel R. Effect of toxin a and B of *Clostridium difficile* on rabbit ileum and colon. Gut. 1986;27(1):78–85. doi:10.1136/gut.27.1.78. 3949240 PMC1433160

[12] Lyerly DM, Lockwood DE, Richardson SH, Wilkins TD. Biological activities of toxins a and B of *Clostridium difficile*. Infect Immun. 1982;35(3):1147–1150. doi:10.1128/iai.35.3.1147-1150.1982. 7068215 PMC351167

[13] Taylor NS, Thorne GM, Bartlett JG. Comparison of two toxins produced by *Clostridium difficile*. Infect Immun. 1981;34(3):1036–1043. doi:10.1128/iai.34.3.1036-1043.1981. 7333662 PMC350971

[14] Moore R, Pothoulakis C, LaMont JT, Carlson S, Madara JL. *C. difficile* toxin a increases intestinal permeability and induces Cl^−^ secretion. Am J Physiol. 1990;259(2):G165–172. doi:10.1152/ajpgi.1990.259.2.G165. 2116728

[15] Mitchell TJ, Ketley JM, Burdon DW, Candy DCA, Stephen J. The effects of *Clostridium difficile* crude toxins and purified toxin a on stripped rabbit ileal mucosa in Ussing chambers. J Med Microbiol. 1987;23(3):199–204. doi:10.1099/00222615-23-3-199. 2856844

[16] Triadafilopoulos G, Pothoulakis C, O’Brien MJ, LaMont JT. Differential effects of *Clostridium difficile* toxins a and B on rabbit ileum. Gastroenterology. 1987;93(2):273–279. doi:10.1016/0016-5085(87)91014-6. 3596162

[17] Das S, Jayaratne R, Barrett KE. The role of ion transporters in the pathophysiology of infectious diarrhea. Cell Mol Gastroenterol Hepatol. 2018;6(1):33–45. doi:10.1016/j.jcmgh.2018.02.009. 29928670 PMC6007821

[18] Hayashi H, Szászi K, Coady-Osberg N, Furuya W, Bretscher AP, Orlowski J, Grinstein S. Inhibition and redistribution of NHE3, the apical Na^+^/H^+^ exchanger, by *Clostridium difficile* toxin B. J Gen Physiol. 2004;123(5):491–504. doi:10.1085/jgp.200308979. 15078917 PMC2234495

[19] Engevik MA, Engevik KA, Yacyshyn MB, Wang J, Hassett DJ, Darien B, Yacyshyn BR, Worrell RT. Human *Clostridium difficile* infection: inhibition of NHE3 and microbiota profile. Am J Physiol-Gastrointest Liver Physiol. 2014;308(6):G497–G509. doi:10.1152/ajpgi.00090.2014. 25552580 PMC4422371

[20] Coffing H, Priyamvada S, Anbazhagan AN, Salibay C, Engevik M, Versalovic J, Yacyshyn MB, Yacyshyn B, Tyagi S, Saksena S, et al. *Clostridium difficile* toxins a and B decrease intestinal SLC26A3 protein expression. Am J Physiol - Gastrointest Liver Physiol. 2018;315(1):G43–G52. doi:10.1152/ajpgi.00307.2017. 29597352 PMC6109705

[21] Janecke AR, Heinz-Erian P, Yin J, Petersen B-S, Franke A, Lechner S, Fuchs I, Melancon S, Uhlig HH, Travis S, et al. Reduced sodium/proton exchanger NHE3 activity causes congenital sodium diarrhea. Hum Mol Genet. 2015;24(23):6614–6623. doi:10.1093/hmg/ddv367. 26358773 PMC4634371

[22] Höglund P, Haila S, Socha J, Tomaszewski L, Saarialho-Kere U, Karjalainen-Lindsberg M-L, Airola K, Holmberg C, de la Chapelle A, Kere J, et al. Mutations of the Down–regulated in adenoma (DRA) gene cause congenital chloride diarrhoea. Nat Genet. 1996;14(3):316–319. doi:10.1038/ng1196-316. 8896562

[23] Gawenis LR, Stien X, Shull GE, Schultheis PJ, Woo AL, Walker NM, Clarke LL. Intestinal NaCl transport in NHE2 and NHE3 knockout mice. Am J Physiol Gastrointest Liver Physiol. 2002;282(5):G776–784. doi:10.1152/ajpgi.00297.2001. 11960774

[24] Schweinfest CW, Spyropoulos DD, Henderson KW, Kim J-H, Chapman JM, Barone S, Worrell RT, Wang Z, Soleimani M. Slc26a3 (dra)-deficient mice display chloride-losing diarrhea, enhanced colonic proliferation, and distinct up-regulation of ion transporters in the colon. J Biol Chem. 2006;281(49):37962–37971. doi:10.1074/jbc.M607527200. 17001077

[25] Clayburgh DR, Musch MW, Leitges M, Fu Y-X, Turner JR. Coordinated epithelial NHE3 inhibition and barrier dysfunction are required for TNF-mediated diarrhea in vivo. J Clin Invest. 2006;116(10):2682–2694. doi:10.1172/JCI29218. 17016558 PMC1578628

[26] Erokhova L, Horner A, Ollinger N, Siligan C, Pohl P. The sodium glucose cotransporter SGLT1 is an extremely efficient facilitator of passive water transport. J Biol Chem. 2016;291(18):9712–9720. doi:10.1074/jbc.M115.706986. 26945065 PMC4850308

[27] Martín MG, Turk E, Lostao MP, Kerner C, Wright EM. Defects in Na^+^/glucose cotransporter (SGLT1) trafficking and function cause glucose-galactose malabsorption. Nat Genet. 1996;12(2):216–220. doi:10.1038/ng0296-216. 8563765

[28] Talbot C, Lytle C. Segregation of Na/H exchanger-3 and Cl/HCO3 exchanger SLC26A3 (DRA) in rodent cecum and colon. Am J Physiol-Gastrointest Liver Physiol. 2010;299(2):G358–G367. doi:10.1152/ajpgi.00151.2010. 20466943

[29] Cha B, Tse M, Yun C, Kovbasnjuk O, Mohan S, Hubbard A, Arpin M, Donowitz M. The NHE3 juxtamembrane cytoplasmic domain directly binds ezrin: dual role in NHE3 trafficking and mobility in the brush border. Mol Biol Cell. 2006;17(6):2661–2673. doi:10.1091/mbc.e05-09-0843. 16540524 PMC1474801

[30] Riegler M, Sedivy R, Pothoulakis C, Hamilton G, Zacherl J, Bischof G, Cosentini E, Feil W, Schiessel R, LaMont JT, et al. *Clostridium difficile* toxin B is more potent than toxin a in damaging human colonic epithelium in vitro. J Clin Invest. 1995;95(5):2004–2011. doi:10.1172/JCI117885. 7738167 PMC295778

[31] Heyman M, Corthier G, Lucas F, Meslin JC, Desjeux JF. Evolution of the caecal epithelial barrier during *Clostridium difficile* infection in the mouse. Gut. 1989;30(8):1087–1093. doi:10.1136/gut.30.8.1087. 2504650 PMC1434188

[32] Chanez-Paredes SD, Abtahi S, Kuo W-T, Turner JR. Differentiating between tight junction-dependent and tight junction-independent intestinal barrier loss in vivo. Methods Mol Biol. 2021;2367:249–271. 33830456 10.1007/7651_2021_389 PMC8249353

[33] Clayburgh DR, Barrett TA, Tang Y, Meddings JB, Van Eldik LJ, Watterson DM, Clarke LL, Mrsny RJ, Turner JR. Epithelial myosin light chain kinase-dependent barrier dysfunction mediates T cell activation-induced diarrhea *in vivo*. J Clin Invest. 2005;115(10):2702–2715. doi:10.1172/JCI24970. 16184195 PMC1224297

[34] Kang L, Fang X, Song Y-H, He Z-X, Wang Z-J, Wang S-L, Li Z-S, Bai Y. Neutrophil–epithelial crosstalk during intestinal inflammation. Cell Mol Gastroenterol Hepatol. 2022;14(6):1257–1267. doi:10.1016/j.jcmgh.2022.09.002. 36089244 PMC9583449

[35] Hecht G, Pothoulakis C, LaMont JT, Madara JL. *Clostridium difficile* toxin a perturbs cytoskeletal structure and tight junction permeability of cultured human intestinal epithelial monolayers. J Clin Invest. 1988;82(5):1516–1524. doi:10.1172/JCI113760. 3141478 PMC442717

[36] Nusrat A, von Eichel-Streiber C, Turner JR, Verkade P, Madara JL, Parkos CA. *Clostridium difficile* toxins disrupt epithelial barrier function by altering membrane microdomain localization of tight junction proteins. Infect Immun. 2001;69(3):1329–1336. doi:10.1128/IAI.69.3.1329-1336.2001. 11179295 PMC98024

[37] Mileto SJ, Jardé T, Childress KO, Jensen JL, Rogers AP, Kerr G, Hutton ML, Sheedlo MJ, Bloch SC, Shupe JA, et al. *Clostridioides difficile* infection damages colonic stem cells via TcdB, impairing epithelial repair and recovery from disease. Proc Natl Acad Sci. 2020;117(14):8064–8073. doi:10.1073/pnas.1915255117. 32198200 PMC7149309

[38] Drewes JL, Chen J, Markham NO, Knippel RJ, Domingue JC, Tam AJ, Chan JL, Kim L, McMann M, Stevens C, et al. Human colon cancer–derived *Clostridioides difficile* strains drive colonic tumorigenesis in mice. Cancer Discov. 2022;12(8):1873–1885. doi:10.1158/2159-8290.CD-21-1273. 35678528 PMC9357196

[39] Marchelletta RR, Gareau MG, McCole DF, Okamoto S, Roel E, Klinkenberg R, Guiney DG, Fierer J, Barrett KE. Altered expression and localization of ion transporters contribute to diarrhea in mice with Salmonella-induced enteritis. Gastroenterology. 2013;145(6):1358–1368.e4. doi:10.1053/j.gastro.2013.08.054. 24001788 PMC3899031

[40] Hodges K, Gill R. Infectious diarrhea: cellular and molecular mechanisms. Gut Microbes. 2010;1(1):4–21. doi:10.4161/gmic.1.1.11036. 21327112 PMC3035144

[41] Lehmann A, Hornby PJ. Intestinal SGLT1 in metabolic health and disease. Am J Physiol-Gastrointest Liver Physiol. 2016;310(11):G887–G898. doi:10.1152/ajpgi.00068.2016. 27012770

[42] Yoshikawa T, Inoue R, Matsumoto M, Yajima T, Ushida K, Iwanaga T. Comparative expression of hexose transporters (SGLT1, GLUT1, GLUT2 and GLUT5) throughout the mouse gastrointestinal tract. Histochem Cell Biol. 2011;135(2):183–194. doi:10.1007/s00418-011-0779-1. 21274556

[43] González Bosc LV, Vidal NA, Prieto R, Tur JA. Effect of atrial natriuretic peptide on α-methyl-d-glucoside intestinal active uptake in rats. Peptides. 1998;19(7):1249–1253. doi:10.1016/S0196-9781(98)00072-2. 9786175

[44] Honka H, Mäkinen J, Hannukainen JC, Tarkia M, Oikonen V, Teräs M, Fagerholm V, Ishizu T, Saraste A, Stark C, et al. Validation of [18F]fluorodeoxyglucose and positron emission tomography (PET) for the measurement of intestinal metabolism in pigs, and evidence of intestinal insulin resistance in patients with morbid obesity. Diabetologia. 2013;56(4):893–900. doi:10.1007/s00125-012-2825-5. 23334481

[45] Antunes A, Martin-Verstraete I, Dupuy B. CcpA-mediated repression of *Clostridium difficile* toxin gene expression. Mol Microbiol. 2011;79(4):882–899. doi:10.1111/j.1365-2958.2010.07495.x. 21299645

[46] Dupuy B, Sonenshein AL. Regulated transcription of *Clostridium difficile* toxin genes. Mol Microbiol. 1998;27(1):107–120. doi:10.1046/j.1365-2958.1998.00663.x. 9466260

[47] Mefferd CC, Bhute SS, Phan JR, Villarama JV, Do DM, Alarcia S, Abel-Santos E, Hedlund BP. A high-fat/high-protein, atkins-type diet exacerbates *Clostridioides* (*Clostridium*) *difficile* infection in mice, whereas a high-carbohydrate diet protects. mSystems. 2020;5(1):e00765–19. doi:10.1128/mSystems.00765-19. PMC7018531 32047064

[48] Engevik AC, Goldenring JR. Trafficking ion transporters to the apical membrane of polarized intestinal enterocytes. Cold Spring Harb Perspect Biol. 2018;10(1):a027979. doi:10.1101/cshperspect.a027979. 28264818 PMC5683927

[49] Kekuda R, Saha P, Sundaram U. Role of Sp1 and HNF1 transcription factors in SGLT1 regulation during chronic intestinal inflammation. Am J Physiol-Gastrointest Liver Physiol. 2008;294(6):G1354–G1361. doi:10.1152/ajpgi.00080.2008. 18339704

[50] Malakooti J, Saksena S, Gill RK, Dudeja P. Transcriptional regulation of the intestinal luminal Na + and Cl − transporters. Biochem J. 2011;435(2):313–325. doi:10.1042/BJ20102062. 21726200 PMC3377368

[51] Sundaram U, Wisel S, Rajendren VM, West AB. Mechanism of inhibition of Na^+^-glucose cotransport in the chronically inflamed rabbit ileum. Am J Physiol-Gastrointest Liver Physiol. 1997;273(4):G913–G919. doi:10.1152/ajpgi.1997.273.4.G913. 9357835

[52] Yu H, Chen K, Sun Y, Carter M, Garey KW, Savidge TC, Devaraj S, Tessier ME, von Rosenvinge EC, Kelly CP, et al. Cytokines are markers of the *Clostridium difficile*-induced inflammatory response and predict disease severity. Clin Vaccine Immunol. 2017;24(8):e00037–17. doi:10.1128/CVI.00037-17. 28592627 PMC5583471

[53] Bruewer M, Luegering A, Kucharzik T, Parkos CA, Madara JL, Hopkins AM, Nusrat A. Proinflammatory cytokines disrupt epithelial barrier function by apoptosis-independent mechanisms. J Immunol. 2003;171(11):6164–6172. doi:10.4049/jimmunol.171.11.6164. 14634132

[54] Kumar A, Priyamvada S, Ge Y, Jayawardena D, Singhal M, Anbazhagan AN, Chatterjee I, Dayal A, Patel M, Zadeh K, et al. A novel role of SLC26A3 in the maintenance of intestinal epithelial barrier integrity. Gastroenterology. 2021;160(4):1240–1255.e3. doi:10.1053/j.gastro.2020.11.008. 33189700 PMC7956241

[55] Engevik AC, Kaji I, Engevik MA, Meyer AR, Weis VG, Goldstein A, Hess MW, Müller T, Koepsell H, Dudeja PK, et al. Loss of MYO5B leads to reductions in Na+ Absorption with maintenance of CFTR-Dependent Cl– Secretion in enterocytes. Gastroenterology. 2018;155(6):1883–1897.e10. doi:10.1053/j.gastro.2018.08.025. 30144427 PMC6279525

[56] Foulke-Abel J, In J, Yin J, Zachos NC, Kovbasnjuk O, Estes MK, de Jonge H, Donowitz M. Human enteroids as a model of upper small intestinal ion transport physiology and pathophysiology. Gastroenterology. 2016;150(3):638–649.e8. doi:10.1053/j.gastro.2015.11.047. 26677983 PMC4766025

[57] Kaji I, Roland JT, Watanabe M, Engevik AC, Goldstein AE, Hodges CA, Goldenring JR. Lysophosphatidic acid increases maturation of brush borders and SGLT1 activity in MYO5B-deficient mice, a model of microvillus inclusion disease. Gastroenterology. 2020;159(4):1390–1405.e20. doi:10.1053/j.gastro.2020.06.008. 32534933 PMC8240502

[58] Ng YK, Ehsaan M, Philip S, Collery MM, Janoir C, Collignon A, Cartman ST, Minton NP. Expanding the repertoire of gene tools for precise manipulation of the *Clostridium difficile* genome: allelic exchange using pyre alleles. Plos One. 2013;8(2):e56051. doi:10.1371/journal.pone.0056051. 23405251 PMC3566075

[59] Kuehne SA, Minton NP. ClosTron-mediated engineering of *Clostridium*. Bioengineered. 2012;3(4):247–254. doi:10.4161/bioe.21004. 22750794 PMC3476875

[60] Otsu N. A threshold selection method from gray-level histograms. IEEE Trans Syst Man Cybern. 1979;9(1):62–66. doi:10.1109/TSMC.1979.4310076.

[61] Theriot CM, Koumpouras CC, Carlson PE, Bergin II, Aronoff DM, Young VB. Cefoperazone-treated mice as an experimental platform to assess differential virulence of *Clostridium difficile* strains. Gut Microbes. 2011;2(6):326–334. doi:10.4161/gmic.19142. 22198617 PMC3337121

[62] Nik AM, Carlsson P. Separation of intact intestinal epithelium from mesenchyme. BioTechniques. 2013;55(1):42–44. doi:10.2144/000114055. 23834385

