# Supplementary figures and images for: Increased intestinal permeability and downregulation of absorptive ion transporters Nhe3, Dra, and Sglt1 contribute to diarrhea during Clostridioides difficile infection
Source: Gut Microbes. 2023 Jun 23;15(1):2225841. doi: 10.1080/19490976.2023.2225841 (PMC10291935; doi:10.1080/19490976.2023.2225841)

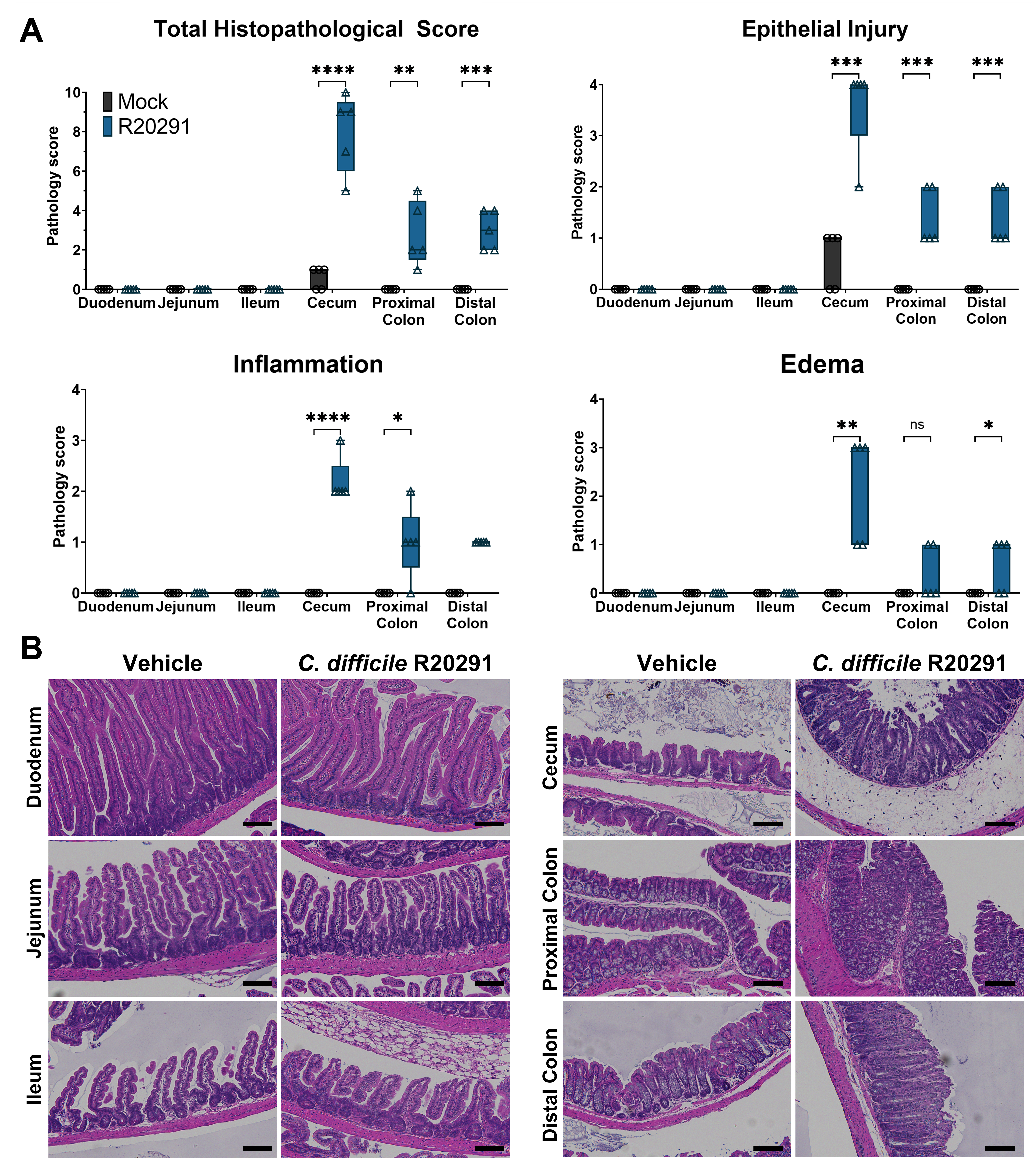

Supplement: Supplemental Material [file KGMI_A_2225841_SM8116.zip › Supplemental data/SupplementalFigure1.png]

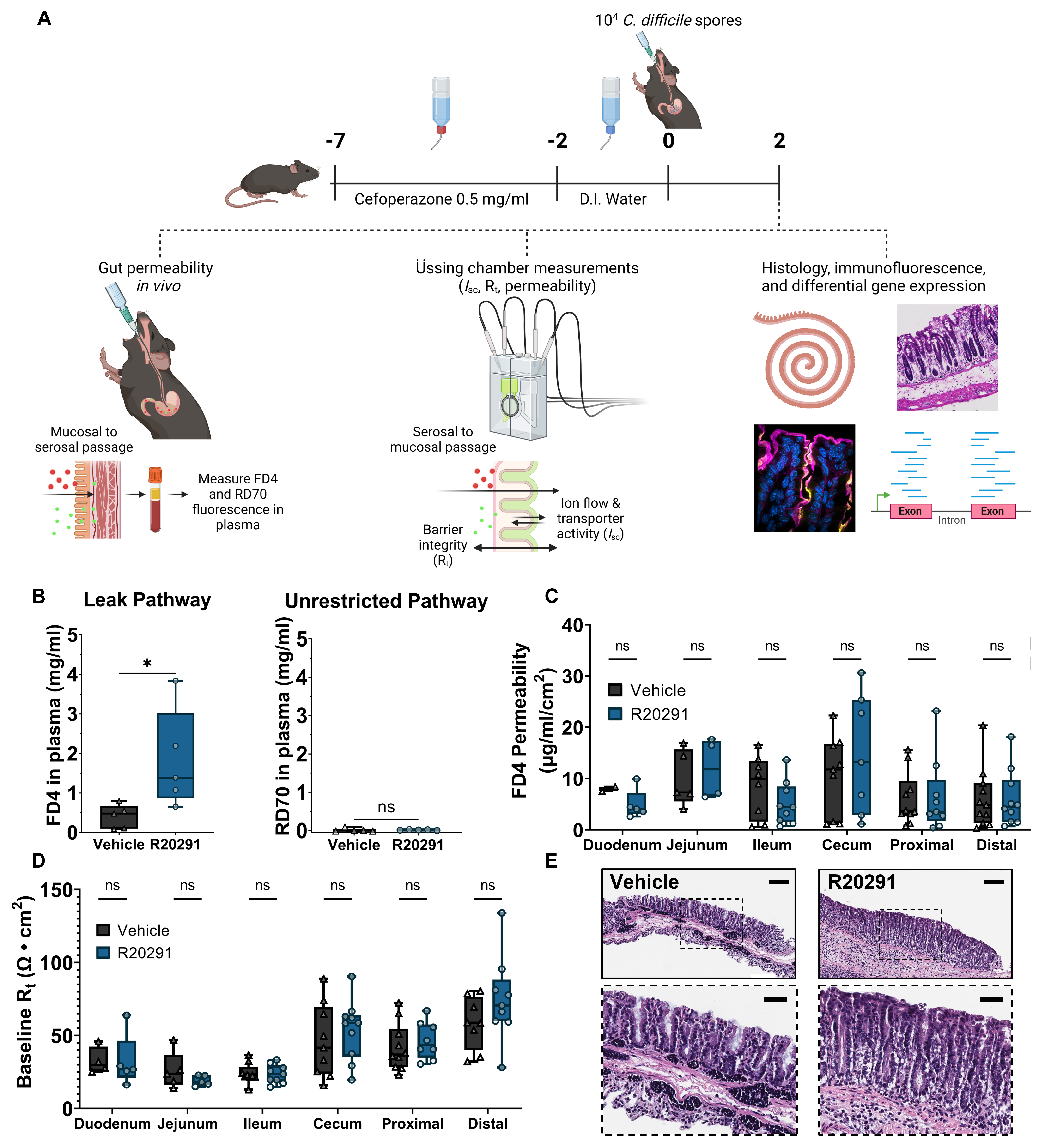

Supplement: Supplemental Material [file KGMI_A_2225841_SM8116.zip › Supplemental data/SupplementalFigure2.png]

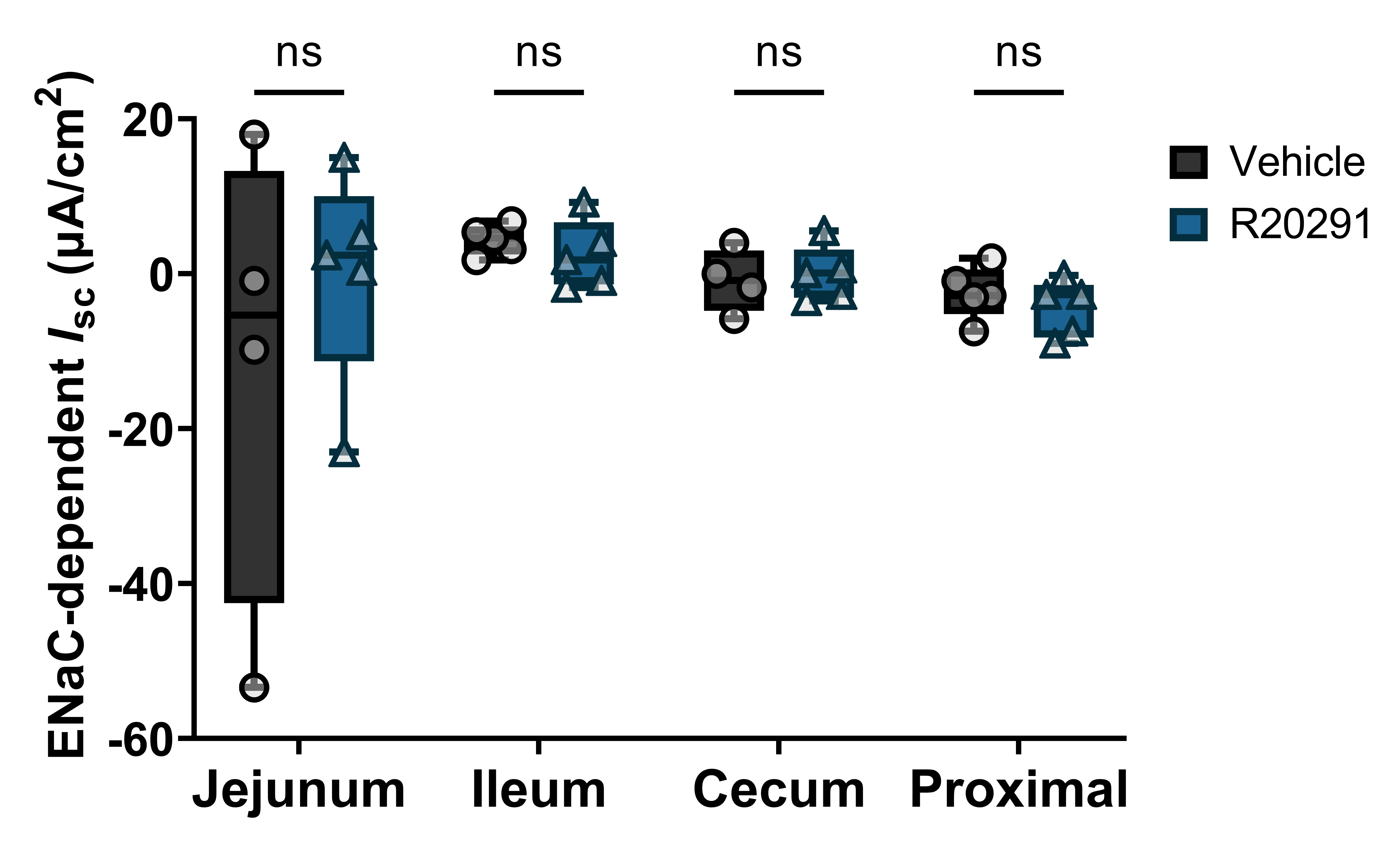

Supplement: Supplemental Material [file KGMI_A_2225841_SM8116.zip › Supplemental data/SupplementalFigure3.png]

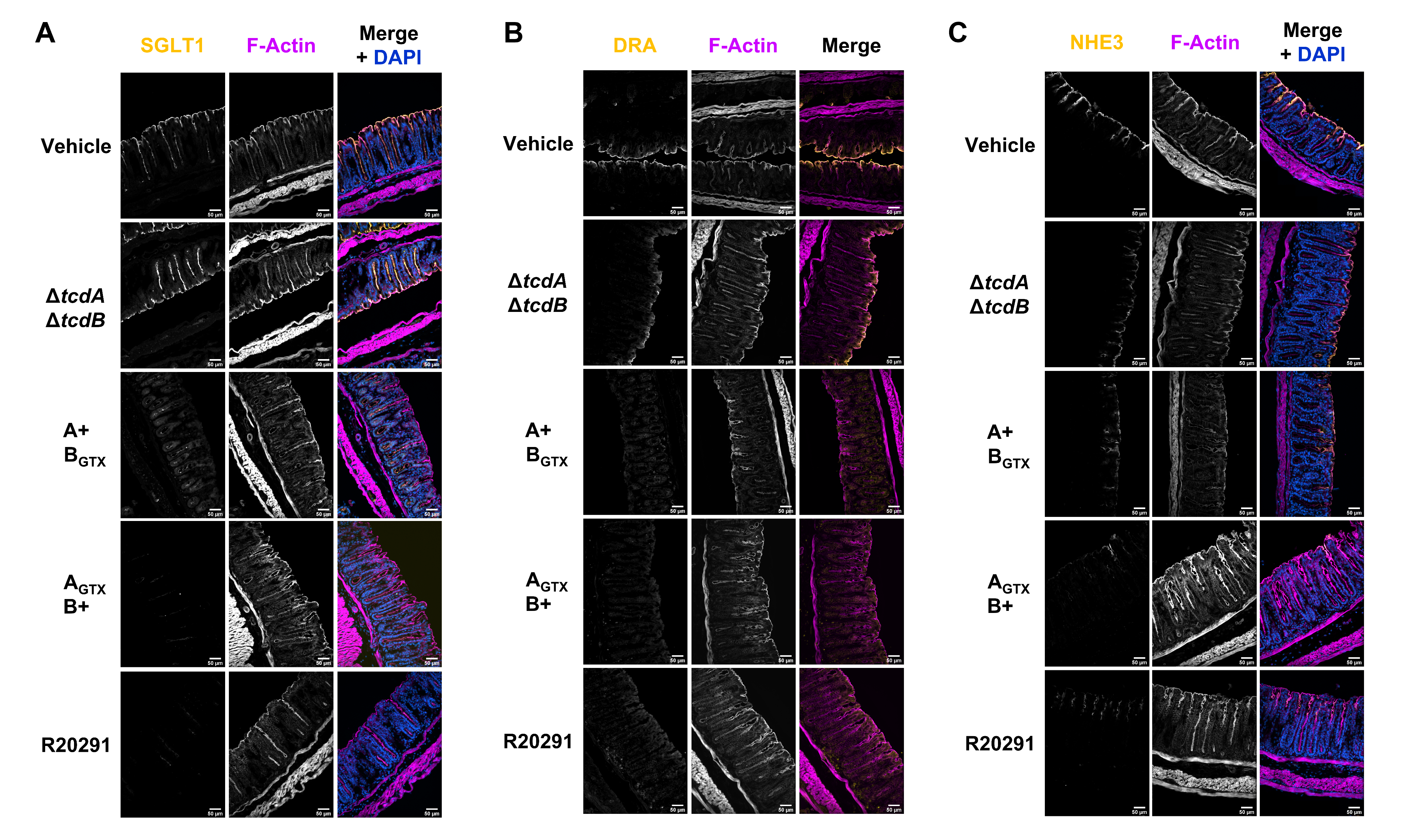

Supplement: Supplemental Material [file KGMI_A_2225841_SM8116.zip › Supplemental data/SupplementalFigure4.png]
